# Rapid consumption of H_2_ in the deep subsurface

**DOI:** 10.1093/nsr/nwag496

**Published:** 2026-08-17

**Authors:** Hao Xie, Ting Xu, Xiaomei Wang, Kun He, Yongbo Peng, Chunlong Yang, Fengtai Tong, Shanyu Han, John M Eiler, Huiming Bao

**Affiliations:** International Center for Isotope Effects Research, State Key Laboratory of Critical Earth Material Cycling and Mineral Deposits, Nanjing University, Nanjing 210023, China; Frontiers Science Center for Critical Earth Material Cycling, School of Earth Sciences and Engineering, Nanjing University, Nanjing 210023, China; International Center for Isotope Effects Research, State Key Laboratory of Critical Earth Material Cycling and Mineral Deposits, Nanjing University, Nanjing 210023, China; Frontiers Science Center for Critical Earth Material Cycling, School of Earth Sciences and Engineering, Nanjing University, Nanjing 210023, China; Research Institute of Petroleum Exploration & Development, PetroChina, Beijing 100083, China; Key Laboratory of Petroleum Geochemistry, China National Petroleum Corporation, Beijing 100083, China; Research Institute of Petroleum Exploration & Development, PetroChina, Beijing 100083, China; Key Laboratory of Petroleum Geochemistry, China National Petroleum Corporation, Beijing 100083, China; International Center for Isotope Effects Research, State Key Laboratory of Critical Earth Material Cycling and Mineral Deposits, Nanjing University, Nanjing 210023, China; Frontiers Science Center for Critical Earth Material Cycling, School of Earth Sciences and Engineering, Nanjing University, Nanjing 210023, China; Research Institute of Petroleum Exploration & Development, PetroChina, Beijing 100083, China; Key Laboratory of Petroleum Geochemistry, China National Petroleum Corporation, Beijing 100083, China; International Center for Isotope Effects Research, State Key Laboratory of Critical Earth Material Cycling and Mineral Deposits, Nanjing University, Nanjing 210023, China; Frontiers Science Center for Critical Earth Material Cycling, School of Earth Sciences and Engineering, Nanjing University, Nanjing 210023, China; International Center for Isotope Effects Research, State Key Laboratory of Critical Earth Material Cycling and Mineral Deposits, Nanjing University, Nanjing 210023, China; Frontiers Science Center for Critical Earth Material Cycling, School of Earth Sciences and Engineering, Nanjing University, Nanjing 210023, China; Division of Geological and Planetary Sciences, California Institute of Technology, Pasadena, CA 91106, USA; International Center for Isotope Effects Research, State Key Laboratory of Critical Earth Material Cycling and Mineral Deposits, Nanjing University, Nanjing 210023, China; Frontiers Science Center for Critical Earth Material Cycling, School of Earth Sciences and Engineering, Nanjing University, Nanjing 210023, China

**Keywords:** natural hydrogen, clumped isotopes, deep hydrogen

## Abstract

Subsurface natural hydrogen is an emerging clean energy resource, but its origins, loss mechanisms and cycling timescales, particularly at greater depths, remain poorly constrained. Here, we report large disequilibrium in the clumped isotopes of H_2_ (^2^H^2^H) and hydrogen isotope fractionation between water and H_2_, measured in gas samples (containing 0.10%–1.59% H_2_) collected from deep (3000–7000 m) wells in the central Sichuan Basin. Because molecular hydrogen can equilibrate isotopes rapidly in the presence of water, such large disequilibrium indicates active kinetically controlled production and/or consumption. Analyses of geological and chemical evidence demonstrate that isotopic disequilibrium is driven by consumption by chemical reactions in host rocks coupled with partial hydrogen exchange with co-existing water. The results imply that measured compositions do not preserve the information on original sources and processes. More broadly, the dynamic cycling of hydrogen renders sediment-hosted deep gas reservoirs unfavorable for long-term or large-scale accumulation of natural hydrogen.

## Teaser

Natural hydrogen is a potential clean energy resource, but isotopic fingerprints from gas wells up to 7 km deep in the Sichuan Basin show that H2 can be consumed by reactions with surrounding rocks on timescales of years. Deep sedimentary reservoirs may therefore be poor places for long-term hydrogen accumulation.

## INTRODUCTION

Molecular hydrogen formed under natural conditions is a target for exploration as an energy resource because of its zero-carbon emissions and potential for large subsurface accumulations [1]. However, the subsurface processes that govern hydrogen formation, accumulation and stability under various geologic conditions are uncertain, hindering our ability to predict locations of economically viable deposits [2,3]. Notably, molecular hydrogen can be generated and lost via multiple chemical and physical mechanisms. Its generation pathways include (i) serpentinization, (ii) water radiolysis, (iii) microbial hydrogenesis and (iv) thermal decomposition of organic matter. Its loss pathways include (i) consumption through abiotic chemical reactions (e.g. hydrogenation of organic matter and hydrogen oxidation by minerals or inorganic gases such as CO_2_) [4–6], (ii) transport loss (e.g. advective fracture flow or diffusion through caprock) [7] and (iii) microbial consumption. These processes control the dynamics of subsurface hydrogen, potentially driving and limiting formation of large natural hydrogen accumulations [1] and will impact any efforts to construct underground hydrogen storage [8].

The origins and fates of natural hydrogen can be constrained through measurements of its hydrogen stable-isotope ratio (δ^2^H_VSMOW_ = (^2^H/^1^H)_sample_/(^2^H/^1^H)_VSMOW_ – 1; VSMOW: Vienna Standard Mean Ocean Water). This approach takes advantage of the fact that H_2_ generation and loss mechanisms involve isotopic fractionations, leading to diversity in ^2^H/^1^H ratio among various sources and residues of various sink processes [2,3]. Additionally, the hydrogen isotope ratio is also influenced by isotope exchange reactions that equilibrate isotope ratios with co-existing water, which is fast (half-life of 10^−1^–10^2^ years) under shallow-crustal temperatures [9,10]. However, in practice the ^2^H/^1^H ratio of H_2_ (or accompanied by ^2^H/^1^H ratio measurements of co-occurring water) may be of limited use for defining sources and sinks of H_2_ in subsurface settings because of the multitude of confounding factors that potentially influence these ratios [11]. Here, we explore the additional constraints provided by measurements in H_2_-rich natural gases of the H_2_ clumped isotope signature (Δ^2^H^2^H = 4^2^H^2^H × ^1^H^1^H/(^2^H^1^H)^2^−1). ∆^2^H^2^H measures the departure of the abundance of the ^2^H^2^H isotopologue from that expected for random distribution of ^2^H among all co-occurring H_2_ molecules, and provides an additional independent constraint on the processes controlling subsurface H_2_ accumulations [12–14].

**Figure 1.**
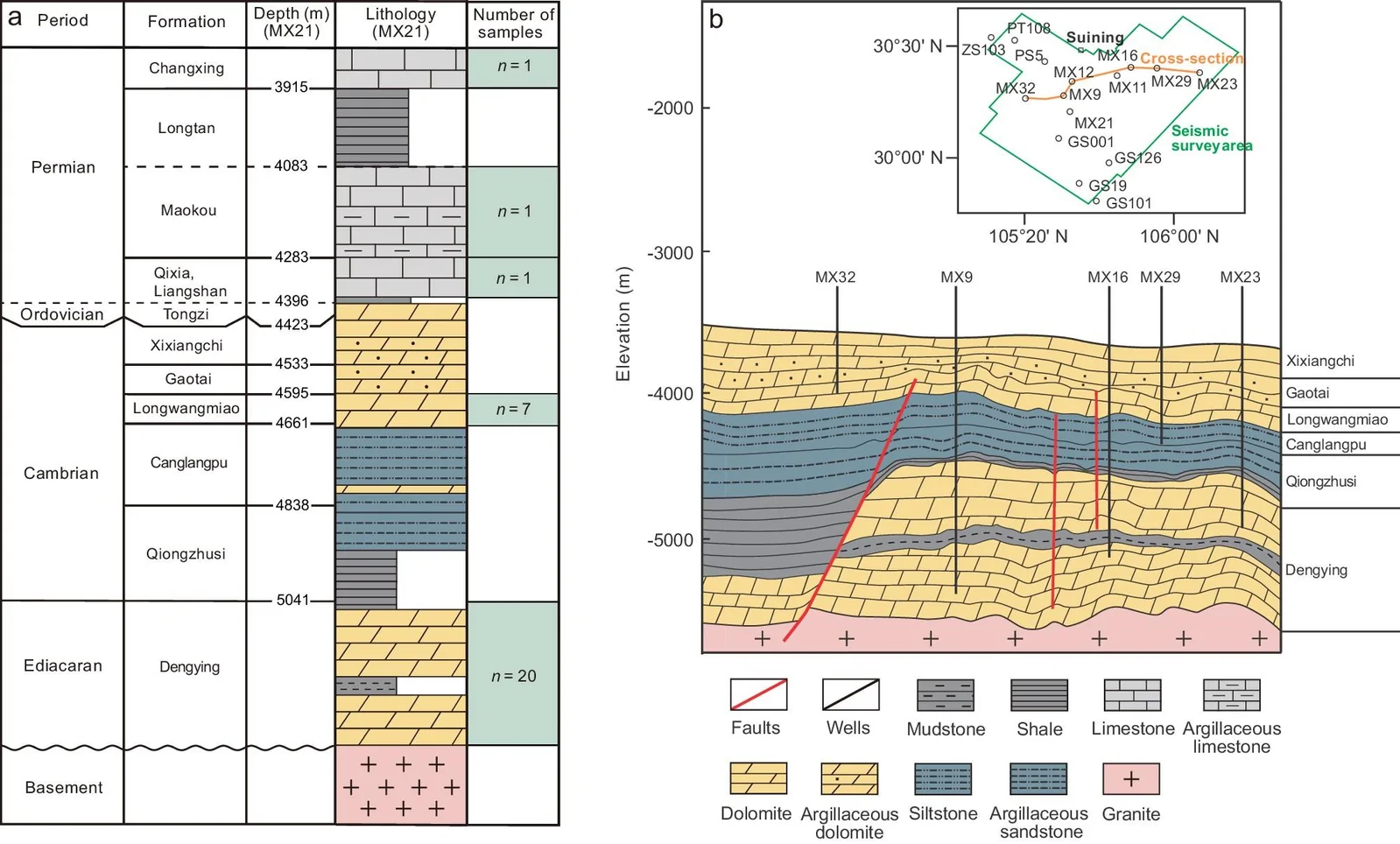
Geological context of the studied area. (a) Stratigraphy for the studied area, with depth model from well MX21. (b) A cross-section produced by integrating 3D seismic reflection data with well log data.

We investigate deep subsurface H_2_ from conventional thermogenic natural gas samples (*n* = 30), collected from production wells in the Sichuan Basin, China, between 2012 and 2023. These gas samples contain 0.10%–1.59% H_2_ (by volume), representing dilute H_2_ in methane-dominated gas. The sampled natural gas reservoir formations span a wide range in depth (between 2939 and 7110 m; median = 5445 m) and depositional age (from Ediacaran to Permian), and have current temperatures of 90–175°C (median = 162°C) at the depths of the sampled boreholes. Their reservoir rocks are dominantly dolomitic, and the basement rocks are granitic (Fig. 1). These samples offer a valuable opportunity to explore hydrogen accumulation and loss dynamics at depth. Conceptual models of natural H_2_ accumulations often call on deep crustal sources to support shallow accumulations discovered through drilling [15,16]; thus, this study examines the dynamics of geological systems that may ultimately support economically useful accumulations.

We extracted molecular hydrogen from natural gas and analyzed its hydrogen-isotope and clumped-isotope compositions alongside previously determined gas compositions and hydrocarbon isotope compositions (see the Methods section and Supplementary Data S1).

## RESULTS AND DISCUSSION

### Isotopic signatures of deep subsurface hydrogen

**Figure 2.**
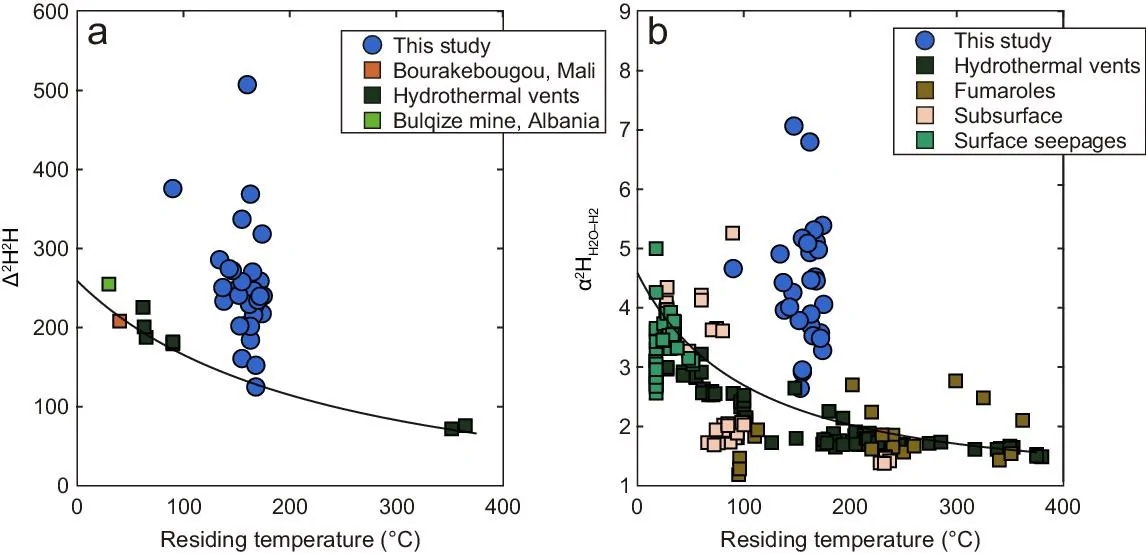
Isotopic composition of molecular hydrogen in gas samples. (a) Clumped isotope composition of H_2_ from natural gas in the Sichuan Basin (this study) and a few previously examined locations [13,14], plotted against their residing temperatures measured at the sample site. The residing temperature refers to the temperature at which the gas is found at the time of sampling, determined either through direct measurement or inferred from geothermal gradients. The solid curve shows theoretical values of thermodynamic equilibrium fractionation, using the path integral Monte Carlo (PIMC) calculations from Korol *et al*. [40]. (b) H_2_O–H_2_ hydrogen isotope fractionation in central Sichuan Basin natural gases (this study) and other origins [27,41–55]. The surface seepage type includes gases found in springs, seeps and outcrop rock samples. The curve is the experimentally calibrated thermodynamic equilibrium fractionation for H_2_O(l)–H_2_(g) from Hochscheid *et al*. [56].

Molecular hydrogen in these samples exhibits high Δ^2^H^2^H values (125–507 ‰), generally exceeding the thermodynamic equilibrium fractionation values corresponding to the borehole temperatures at which they were collected (logged at the time of sampling; Fig. 2a). This pronounced clumped isotope disequilibrium contrasts with previously analyzed molecular hydrogen samples from the Bourakebougou area in Mali and hydrothermal vents along the Atlantic mid-ocean ridges, where clumped isotope equilibrium under present-day environmental temperatures has been reached or closely approached [13].

Sichuan Basin gases are also characterized by low δ^2^H_VSMOW_ values of H_2_ (−866−641 ‰) compared with most natural samples [3]. Because formation waters associated with our studied samples are within the common range for basinal fluids (we take a value of δ^2^H_VSMOW_ = −50 ‰ based on prior analyses of water from the formations studied here [17]), it follows that ^2^α_H2O–H2_ (^2^α_H2O–H2_ = (^2^H/^1^H)_H2O_/(^2^H/^1^H)_H2_) values for our samples are unusually high—exceeding equilibrium values at the corresponding temperatures by factors of 1.2 to 3.1 (median = 1.9). By comparison, published ^2^α_H2O–H2_ for other natural samples of hydrogen often equal or fall below values corresponding to equilibrium corresponding to their residing temperatures (those samples that fall below equilibrium ^2^α_H2O–H2_ values have been suggested to reflect incomplete isotopic exchange between H_2_ and H_2_O during migration and cooling of the fluid [10]). Similarly, the Sichuan Basin hydrogen isotope fractionation between co-existing methane and H_2_ (^2^⍺_CH4–H2_ = (^2^H/^1^H)_CH4_/(^2^H/^1^H)_H2_) also exceeds that predicted for equilibrium at corresponding temperatures by factors of 0.9 to 2.6 (median 1.5; Fig. S1).

Thus, taken together, measurements of Δ^2^H^2^H, ^2^α_H2O–H2_ and ^2^α_CH4–H2_ in Sichuan Basin gases indicate pronounced and near ubiquitous isotopic disequilibrium with respect to all isotope equilibria involving H_2_. Importantly, H_2_ is known to exchange and equilibrate its isotopes with water relatively quickly (half-life = 5.7 years at 100°C in the presence of saturated water vapor [10]). Calculations suggest that the maximum dwell times (longest times since the imposition of isotopic disequilibrium in H_2_) are below 20 years for all samples, with most falling below 1 year (see Data S1 and SI Text of the Supplementary Information). We conclude that the isotope disequilibria we observe indicate that active or recent chemical and/or physical processes modified the isotopic compositions of H_2_. Most such processes (detailed below) also modify H_2_ concentration, suggesting assays for geological H_2_ in deep drill holes may not reflect true long-term average concentrations in the respective host rocks. This finding contrasts with the assumptions underlying some recent discussions of well-gas data for geological H_2_ deposits, which generally relate observations of collected gas directly to H_2_ generation processes at depth [3,18]. Any interpretations regarding the expected long-term average concentrations or lifetimes of H_2_ in the Sichuan Basin source rocks can be made only with an understanding of the processes driving the recent dynamic processing of H_2_ in collected well gases.

### Origin of isotopic disequilibrium

The first question raised by our observations is whether recent dynamic processing of H_2_ in the recovered Sichuan Basin gases is a consequence of the anthropogenic disturbances of drilling, gas extraction and storage, or instead reflects ongoing natural processes at depth. We first consider the former option before turning to the question of natural H_2_ cycling at depth.

**Figure 3.**
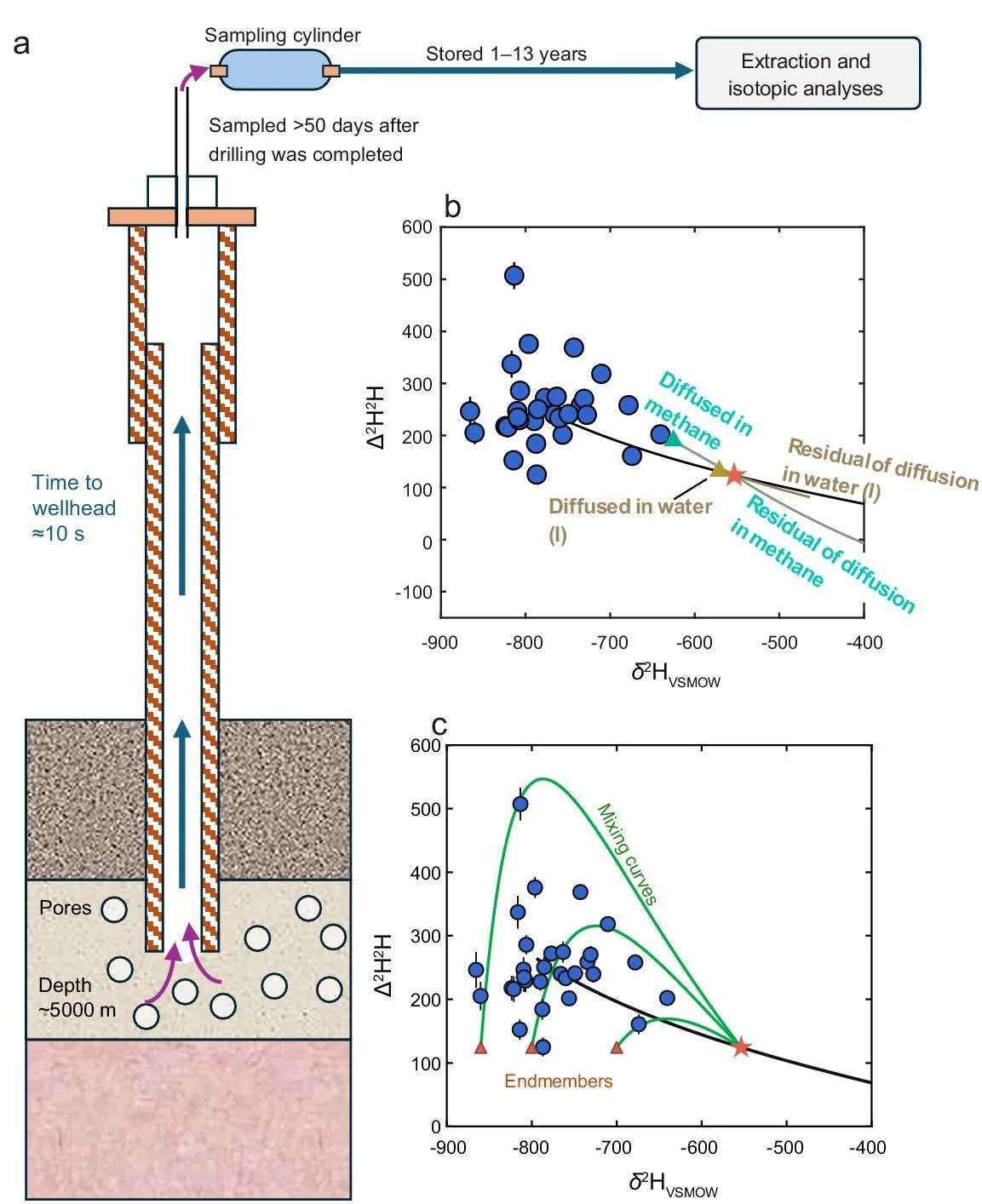
Examination of anthropogenic processes in the central Sichuan Basin samples that may induce isotopic fractionation. (a) Illustration of drilling–production–analyses timescales. (b) Effects of diffusion isotope fractionation associated with the production and sampling process, compared with observed data (see section entitled ‘Methods’). (c) Effects of mixing between gases with hypothetical endmembers that differ significantly in δ^2^H_VSMOW_ values.

It is possible for H_2_ to have been produced, consumed, and/or undergone isotopic exchange during sampling from production natural gas wells and/or during the subsequent 1–13 years of storage in passivated stainless-steel containers (Fig. 3). Anthropogenic H_2_ can be produced during drilling by heating and friction when the drill bit interacts with H-bearing materials [19,20]. We think it implausible that a significant amount of H_2_ of this origin could be in our samples because they were taken from gas-producing wells 50 days or more after drilling completed (except for one sample; see Data S1 of the Supplementary Information). This time span should allow sufficient flushing of any drilling produced gas, as it is six orders of magnitude higher than the typical residence time of gas in the borehole during production gas flow conditions (∼10^−4^ days; see SI Text of the Supplementary Information).

It is possible that migration of H_2_ from source rocks into the well boreholes was controlled by molecular diffusion, which is associated with isotopic fractionation. However, the ‘diffused gas’ endmember calculated for both H_2_ in methane and H_2_ in liquid water is a poor quantitative match for the measured δ^2^H_VSMOW_ and Δ^2^H^2^H values of our samples (Fig. 3; see SI Text of the Supplementary Information for detailed calculations and additional scenarios). We thereby conclude that the isotopic fractionations associated with gas migration cannot explain our findings, though migration effects could contribute as part of a more complex, multifactor process driving isotopic disequilibrium. This conclusion revises the previous interpretation of the same sample set by Wang *et al*. [21], which identified diffusion-driven fractionation as the most likely mechanism based solely on δ²H_VSMOW_ values. The revised interpretation highlights the additional constraints and diagnostic power provided by clumped isotope analyses.

Drilling and gas production may result in mixing of different fluids, which produces positive anomalies in the Δ^2^H^2^H values if endmembers differ in hydrogen isotopes, as previously described in clumped isotope systems [13,22,23]. We can constrain the isotopic properties of potential endmembers required to explain the observed data. To account for the range of Δ^2^H^2^H and δ^2^H_VSMOW_ signatures in Sichuan Basin gases, the low δ^2^H_VSMOW_ endmember would have δ^2^H_VSMOW_ < −860 ‰ (Fig. 3 and Fig. S2). Such value is far from isotopic equilibrium and does not fit known hydrogen sources (generally >−800 ‰; [3]). We do not fully exclude the existence of an ultra-depleted H_2_ because natural H_2_ with δ^2^H values below −900‰ have been reported in a rare case [18]. However, simple mixing remains an incomplete explanation for the Sichuan data because it cannot account for the origin of the low δ^2^H endmember. We, therefore, regard mixing as a possible contributing process, but not the dominant cause of the observed isotope disequilibrium.

Other anthropogenic factors that might have caused the isotopic disturbances we observe include isotopic exchange at lower temperature, H_2_ generation from corrosion reactions and drilling-induced fluid mixing. We discuss these processes in the SI Text of the Supplementary Information and conclude that they are unlikely drivers of isotopic disequilibrium. None of the anthropogenic processes associated with drilling and gas extraction appear capable of explaining our findings, so our observations must largely or entirely reflect the compositions of well gases prior to their collection. Next, we consider natural generation and loss processes of H_2_, turning over the pool of H_2_ on timescales of years or less, that may cause isotope disequilibrium.

First, the observed disequilibrium signatures may reflect contributions from microbial metabolisms. Arguments in favor of this hypothesis include the ubiquity of H_2_-producing microbial organisms, such as fermenting bacteria, in subsurface environments [24] and experimental indication of low δ^2^H_VSMOW_ values in biogenic H_2_ [25]. However, our samples were taken from well depths where temperatures mostly exceed the upper temperature limit for life (∼122°C [26]); thus, it would appear to be impossible for our results to reflect a microbial origin of the sampled H_2_.

Second, abiotic generation of H_2_ may result in the isotopic disequilibrium. The most typical abiotic sources of H_2_ include water-rock reactions such as serpentinization or through radiolysis of water. However, H_2_ collected from active serpentinization sites, such as hydrothermal vents, typically exhibits δ^2^H_VSMOW_ and ∆^2^H^2^H values approaching or equaling equilibrium controlled by ambient H_2_O and temperature (Fig. 2) [13,27]. Moreover, laboratory experiments have shown that radiolytic H_2_ has a hydrogen isotope fractionation with respect to co-occurring water (^2^α_H2O–H2_) of ∼2 [28,29], far lower than the values we observe (the ∆^2^H^2^H value of radiolytic H_2_ is presently unknown). Therefore, generation pathways cannot account for the low δ^2^H_VSMOW_ and high ^2^α_H2O–H2_ in the Sichuan Basin gases.

Third, we examine natural loss processes. Physical leakage via diffusion would drive the remaining gas toward higher δ^2^H_VSMOW_ and lower ∆^2^H^2^H, which is opposite to our samples’ distribution (Fig. 3), so diffusion can be excluded as the primary cause of isotopic disequilibrium. Finally, the remaining group of kinetic processes that has not been excluded is abiotic H_2_ consumption, such as through oxidation by ferric oxide minerals, sulfate minerals, pyrite, organic matter and/or CO_2_ (via Fischer–Tropsch-type synthesis). The kinetic isotope effects (KIEs) of these reactions may drive H_2_ away from equilibrium, yet they have not been experimentally or theoretically constrained to our knowledge. We, therefore, performed theoretical calculations of the KIE for several potential oxidants. SO_4_^2−^ and HSO_3_^−^ are selected to represent the oxidation by sulfate minerals; methyl and toluene radicals are selected to represent oxidation by organic compounds. We used density functional theory and statistical mechanical theorem [30] to calculate KIE (see section entitled ‘Methods’). Our calculations show that reacting with toluene or methyl radicals drives the residual H_2_ toward lower Δ^2^H^2^H and higher δ^2^H_VSMOW_ values, opposite to the pattern observed in Sichuan Basin gas samples. In contrast, oxidizing by sulfate or sulfite ions drives residual H_2_ in the same direction as the natural sample data display, although it raises Δ^2^H^2^H too quickly (Fig. S3). However, simultaneous isotopic exchange can help moderate the enrichment of Δ^2^H^2^H.

**Figure 4.**
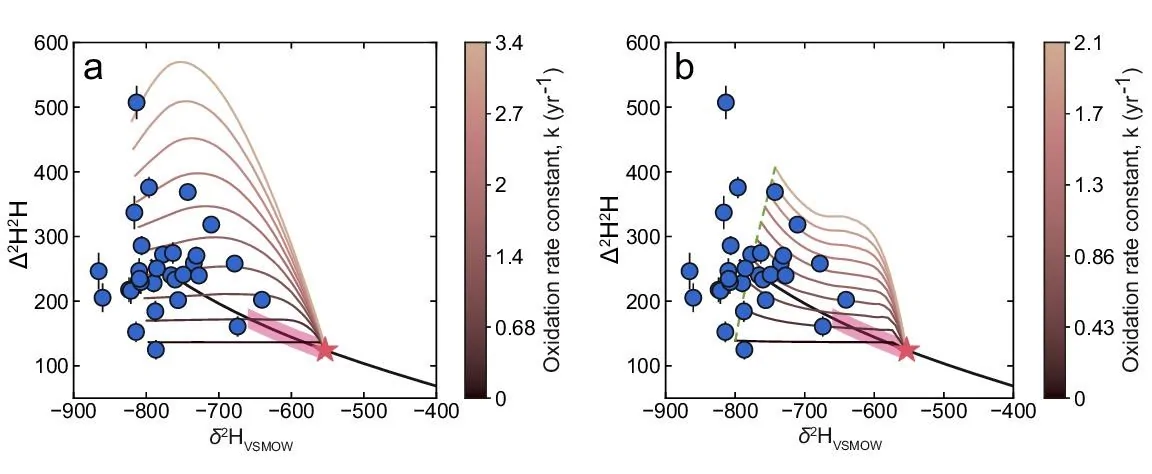
Results of the oxidation-exchange isotope model. (a) Results from the closed-system model. Curves are the results of an oxidation-exchange model, color-coded by pseudo-first order rate constants for hydrogen oxidation. In both panels, the curve is theoretical value of thermodynamic equilibrium fractionation, where Δ^2^H^2^H values are obtained from the anharmonic PIMC calculations from Korol *et al*. [40] and δ^2^H_VSMOW_ values are calculated based on experimentally calibrated α_H2O–H2_ equilibrium from Hochscheid *et al*. [56] and δ^2^H_H2O_ = −50 ‰. In both panels, the band represents equilibrium isotopic composition corresponding to the range of current reservoir temperatures, and the pentagram is the assumed initial isotopic composition. Parameters for initial water and H_2_ conditions can be found in the SI Text of the Supplementary Information. (b) Results from the open-system model. We initialize water at 5 MPa with δ^2^H_VSMOW_ = −50 ‰, and H_2_ at 5 MPa assuming isotope equilibrium with water at 178°C. We assume input flux at 1 MPa yr^−1^ and output rate of 10^−4^ yr^−1^. The dashed line represents steady states.

We model the isotopic evolution of H_2_ assuming oxidation by sulfate is the main loss mechanism and compare results with Sichuan Basin gases. We evaluate such systems with either both H_2_ supply and loss fluxes (open system), or only the loss flux (closed system). Both systems can produce results matching part of the observed data while the closed-system model better reaches the lower δ^2^H_VSMOW_ values observed in many Sichuan samples (Fig. 4). This model result does not require that all wells are hydraulically closed. Instead, it indicates that many samples behaved as loss-dominated systems over the timescale recorded by the H_2_ isotope compositions.

To evaluate where this assumption is most appropriate, we classified the sampled wells by structural domain and available geological evidence for fault/fracture connectivity (Table S6). Most Gaoshiti-Moxi wells occur on relatively low-amplitude, weakly faulted structural highs, and available geological reports do not indicate direct penetration of major faults; these wells are therefore better approximated as closed or weakly open systems. In contrast, wells from the Penglai/Anyue rift-margin domain may have greater potential to be open-system through fluid exchange via existing fault-related conduits. Because direct quantitative constraints such as fault distance, image-log fracture density and repeated time-series sampling are not available for all wells, we use the open- and closed-system models as endmember scenarios rather than assigning an unambiguous binary state to each sample. Interestingly, the hydrogen content of our samples declines with sampling date (Fig. S4), showing a sharp decrease over the past decade that is consistent with the closed-system scenario. Yet, because the samples come from different wells and formations, variations in initial H_2_ content, oxidant availability and water-H_2_ isotope exchange can influence the trend in Fig. S4.

The samples falling outside the predictions may reflect additional isotopic fractionation driven by physical processes such as gas diffusion, alternative oxidation mechanisms or inaccurately estimated initial δ^2^H_VSMOW_ value of H_2_. We test the model’s response to variations of key parameters and find that it is generally insensitive to parameter changes and consistent with observed data (see SI Text and Figs S5–S8 of the Supplementary Information).

Monte Carlo simulations constrain that the oxidation rate constants of Sichuan Basin samples range from 0.015 to 3.5 yr^−1^ (corresponding to half-lives from 0.2 to 47 years) with a median of 1.1 yr^−1^. These rates can be compared with experimentally constrained kinetic rate constants of hydrogen thermochemical sulfate reduction (TSR). An earlier study conducted aqueous TSR experiments between Na_2_SO_4_ and H_2_ at 250 and 280°C [31]. By extrapolating their results to 90–175°C, we obtain second-order kinetic rate constants of 1.4 × 10^−11^–1.5 × 10^−8^ mol^−1^·L·s^−1^, which correspond to H_2_ half–lives of 15–1.5 × 10^4^ years assuming 0.1 M SO_4_^2−^. Although the extrapolated rates are slower than required for our data, the difference may be reconciled by catalysis and heterogeneous gas–solid reactions. TSR reactions have been known to be catalyzed by sulfur intermediate species and heterogeneous mineral surfaces [32,33], which are widely present in the gas reservoir rocks. Additionally, part of the discrepancy may reflect uncertainties associated with extrapolating from high-temperature experiments to the lower temperatures of our system, especially since the two data points were very close. This highlights the need for future experiments that directly constrain H_2_ oxidation kinetics at reservoir-relevant temperatures.

The abiotic oxidation hypothesis is supported by the abundant availability of oxidants and by existing evidence for organic TSR. First, gypsum-bearing sequences are widely deposited in the Cambrian and Triassic periods in the central Sichuan Basin, providing ample reactants for TSR [34,35]. Using the stoichiometric ratio, complete oxidation of H_2_ in 1 m^3^ gas containing 0.10–1.59 mol% H_2_ requires only 1.1–17 g of SO_4_^2−^. Therefore, the 100-m-thick gypsum intervals could have supplied sufficient sulfate to consume the observed H_2_ pool. Although most of our samples reside in dolomite reservoirs (Fig. 1), their production water contains grams-per-liter levels of sulfate concentration (Table S1). Second, multiple chemical and isotopic signs of organic TSR have been reported for the wells and formations examined in this study. Such signs include high δ^34^S_VCDT_ values of H_2_S, pyrite and solid bitumen, and elevated sulfur/carbon (S/C) ratios in organic matter [36–38]. Collectively, these observations and analyses support the plausibility of active hydrogen TSR at depth in the central Sichuan Basin.

## CONCLUSION

Isotopic signatures of molecular hydrogen from the deep Sichuan Basin exhibit significant disequilibrium, consistent with active hydrogen consumption via sulfate-driven oxidation. By combining theoretical predictions of isotope effects with established isotope-exchange rate constants, we estimate that molecular hydrogen is consumed on timescales of years. These rate estimates are very high, exceeding the rate constants of physical loss (<1 × 10^−4^ per year [39]). However, these theoretical results should be interpreted with caution, as potential methodological limitations, such as uncertainty in constraining reaction pathways, may affect the inferred isotope effects. Therefore, these constraints warrant experimental verification. Additionally, we did not cover every oxidant and reaction mechanism, leaving open the possibility of additional oxidation pathways, such as reactions with pyrite and organic functional groups. Nevertheless, the pronounced isotopic disequilibrium indicates that hydrogen loss and turnover must have occurred at comparably rapid rates, regardless of the specific mechanisms involved.

Our study identifies one preservation-risk setting for natural H_2_: deep, hot, hydrocarbon-bearing sedimentary reservoirs that contain reactive oxidants. In this setting, H_2_ can behave as a transient component that is rapidly consumed or isotopically overprinted, rather than as a stable accumulated gas phase. This is distinct from high-H_2_ systems such as Bourakebougou in Mali and hydrothermal/serpentinization sites, where H_2_ can accumulate or persist where generation, migration, trapping and preservation are favorable (Table S7). This finding challenges the prevailing view that low-permeability salt layers serve as effective traps and therefore preferred targets for natural hydrogen exploration [15]. The low H_2_ abundances of these samples (0.10%–1.59%) reflect the result of this extensive consumption. Likewise, a previous survey on samples throughout Australia has found that natural gases tend to have low H_2_ concentrations, with 80% of the samples containing <0.1 mol% H_2_ [18]. Hence, our results provide a straightforward explanation for low H_2_ concentrations observed in sedimentary basins. Future exploration should therefore focus on systems where H_2_ generation, migration and trapping exceed both abiotic and biotic consumption. In particular, deep/hot reservoirs containing sulfate, ferric minerals, pyrite, CO_2_ or reactive organic matter may promote rapid abiotic H_2_ loss, whereas shallow/cool systems must also be evaluated for microbial H_2_ consumption.

## METHODS

The experimental and theoretical methods supporting this study are detailed in the Supplementary Information, which include natural gas sampling techniques, H_2_ preparation protocols and the analytical frameworks employed for isotopic measurements on the Ultra mass spectrometer. It further includes comprehensive sections on analytical error analysis, calibration of isotope analysis via exchange experiments and the derivation of KIEs through the transition state theory. The Supplementary Information text also details the configuration of the oxidation-exchange models and their associated kinetic parameters, alongside sensitivity analyses for the parameters and initial conditions used.

## Supplementary Material

nwag496_Supplemental_Files

## Data Availability

All data are available in the main text or the supplementary information.
