# Supplementary material for: Rapid consumption of H2 in the deep subsurface
Source: Natl Sci Rev. 2026 Aug 17;13(17):nwag496. doi: 10.1093/nsr/nwag496 (PMC13552062; doi:10.1093/nsr/nwag496)
Supplement: Supplementary file 1 [file nwag496_supplemental_files.zip › SI_nsr_hydrogen_r2_final.pdf]

Supplementary Information for

Rapid Consumption of H2 in Deep Subsurface

Hao Xie *et al.*

*Corresponding author. Email: hxie@nju.edu.cn, wxm01@petrochina.com.cn

This PDF file includes:

Supplementary Text
References
Figs. S1 to S21
Tables S1 to S7

Other Supplementary Information for this manuscript include the following:

Data S1 to S5

Supplementary Text

Gas samples

30 natural gas samples were collected at the well locations using stainless-steel cylinders with two-sided needle valves. These cylinders were connected to the gas outlet at the well and flushed with natural gas. Gas pressure in these cylinders is between 1.0 and 5.0 MPa. Chemical composition and carbon isotopes of methane have been analyzed and reported in an earlier study [1]. These properties are tabulated in Data S1. Descriptively, these are dry gas with high methane contents, as their dryness ratio, $C_1/(C_2+C_3)$, is between 80 and 4,800. The carbon isotopes of methane ($\delta^{13}C$) range from -36.7 to -29.1 ‰. These gas compositions and methane carbon isotopes are characteristic of gas from middle to high thermal maturity source rock. Many of these gas samples also have H_2S content (median = 0.8 %). Their helium isotope ratios, expressed as ratio between $^3He/^4He$ of sample and of atmospheric helium (R/Ra), are lower than 0.1 for these samples [1], indicateing higher contribution from crustal helium.

These samples are sourced from the Penglai and Anyue gas fields located in the central Sichuan Basin. Except for three, these samples are derived from the Upper Ediacaran (Sinian) Dengying Formation and the Lower Cambrian Longwangmiao Formation. The Sinian–Cambrian natural gas system in the Sichuan Basin are one of the largest in China and was discovered relatively recently since 2013 [2].

We present a stratigraphic column and a geological cross-section (Fig. 1) to illustrate the geological context of the study area. The stratigraphic column is derived from well log data from one of our sampling wells, MX21. The cross-section was constructed by PetroChina for the region near our sampling sites. It was synthesized by integrating 3D seismic reflection data acquired within the area outlined in green in Fig. 1b with well log data. Seismic data processing included standard workflows such as velocity analysis and normal moveout correction, followed by time-to-depth conversion. Reflectors corresponding to major geological horizons were identified and correlated across seismic sections, and faults were interpreted based on reflector offsets, terminations, and discontinuities. Well log data from multiple wells in the region were integrated with the seismic interpretation to constrain stratigraphic boundaries and construct the final cross-section.

Gas preparation

Natural gas samples in our study contain low concentration of H_2 (0.10–1.59 %), while clumped isotope analysis requires >99.5 mol% pure H_2 gas. We remove all other gas components in natural gas and retrieve H_2 near quantitatively on a gas vacuum line (Fig. S9). First, gases are passed through a trap I (a U-trap) immersed in liquid nitrogen (-196 °C), to remove compounds condensable at this temperature. The gas is then passed through trap II, another U-trap filled with around 2.6 g of Hayesep D porous material (60–80 mesh, Restek), immersed in liquid nitrogen. Trap II can remove all compounds except for helium and H_2. After equilibration of gas pressure for about 1 minute, the residual gas is trapped in trap III, a U-trap filled with 2.7 g of molecular sieve (5A, Sigma-Aldrich), cooled to -196 °C. At this temperature, H_2 can be trapped in molecular sieve near completely with a residual pressure smaller than 0.02 torr. We wait for the gas to transfer for about 3 minutes, then pump down the molecular-sieve trap to remove helium, which impedes the residual H_2

to move towards the molecular-sieve trap. In practice, we close the valve between the trap II and trap III (to prevent methane desorption from the trap II) and vacuum the molecular-sieve trap to remove helium for about 15 seconds. We reopen the valve between the trap II and the molecular-sieve trap, and repeat this transfer-pumping cycle as described. Typically, this cycle is repeated for several times (5–15) until gas pressure from trap II is indistinguishable from that of trap III. Before starting a batch of experiments, both trap II and trap III have been heated under vacuum at 200 °C overnight to activate the Haysep D and molecular sieve. After collecting each sample, trap II and trap III are heated under vacuum at 150 °C to desorb residual gases.

Next, we transfer H_2 from the molecular-sieve trap to a sample glass finger containing about 0.3g of 5A molecular sieve (Sigma-Aldrich) by connecting it to a sample tube cooled in liquid nitrogen and warming the molecular-sieve trap to room temperature. This glass sample tube is then used for mass spectrometry (MS) analysis. Before starting a batch of experiments, every sample tube has been heated under vacuum at 300 °C for 4 hours or longer to activate molecular sieve. Before collecting each sample, the sample tube is heated under vacuum at 300 °C for 10 minutes to desorb residual gases.

Recovery tests are conducted to examine the yield and potential isotopic fractionation during purification. We prepare a mixture with CH_4:H_2 = 20:1 (volume) and processed it using the preparation method above. We use barometric results to determine the yield to be 93 ± 3 % (1 standard deviation; n = 3). We compare isotopic values for one purified sample with its original gas, while results indicate that the changes in hydrogen and clumped isotopes are negligible (Table S2).

Because natural gas samples have a different composition than the mixture used in the purification test, we further evaluated its yield by comparing the amount of H_2 recovered after purification with the expected amount of H_2 calculated from the processed gas volume and the independently measured H_2 concentration of each sample (Data S1). We found >90% yield for samples with low H_2 concentration, comparable to the recovery obtained in the prepared CH_4-H_2 mixture. These high recoveries indicate small and reproducible H_2 loss in the processing of natural samples, such that isotope shifts should be minimal.

Isotopic analysis

Isotopic analysis of molecular hydrogen is performed on a Thermo Scientific Ultra Mass Spectrometer housed at Nanjing University. First, a glass finger storing the sample are heated at 100 °C for 10 mins and introduced to a bellow in the dual-inlet system of the Ultra mass spectrometer. We then examine for ion intensities of ^{4}He, CH_4, H_2O, N_2, O_2, Ar and CO_2 to evaluate concentration of these contaminants. We consider a H_2 gas sample to be clean if the ratios— [intensity of contaminant]/[intensity of H_2]—are smaller than 0.5% for every contaminant except ^{4}He. Note ionization and transmission efficiencies can differ between molecules, such that this ratio cannot be directly treated as abundance ratios. We performed mixing experiments to show that the threshold of 0.5 % intensity ratio corresponds to 0.2 % abundance ratio for N_2/H_2 and 0.7 % abundance ratio for O_2/H_2 (Fig. S10).

^{4}He requires special attention because it has the same cardinal mass as ^{2}H^{2}H but lower than ^{2}H^{2}H by 0.0256 amu. Because the abundance of ^{2}H^{2}H is very low (about 2×10^{-8} for gases with ^{2}H/^{1}H close to VSMOW and 1×10^{-9} for most gases analyzed in this study), scattered ions of ^{4}He ($^4He_{tail}$) might be a non-negligible contribution to the registered intensity at the

measurement position of $^{2}H^{2}H$ (Fig. S11). This contribution might need to be corrected for samples with relatively abundant ^{4}He. The ratio between the tail at the mass of measurement and the ^{4}He peak has been monitored frequently (>2 times per week) and typically is around 1×10^{-7}. Using this ratio, we correct our analyses by subtracting $^{4}He_{tail}/^{1}H^{1}H$ from $^{2}H^{2}H/^{1}H^{1}H$, where $^{4}He_{tail}/^{1}H^{1}H = (^{4}He/^{1}H^{1}H)\times(^{4}He_{tail}/^{4}He)$. We found that this correction is negligible for most of our sample analyses (the size of correction is smaller than the analytical uncertainty), as our samples have been purified to have little helium content. However, it can be significant for samples with $^{4}He/^{1}H^{1}H>1\times10^{-5}$.

The voltages for electronics on the Ultra are optimized to maximize transmission, mass resolving power and the ratios of $^{2}H^{1}H/^{1}H_{3}$ and $^{2}H^{2}H/^{2}H^{1}H_{2}$, where the denominators are hydrogen adducts. These adducts are close in mass to the isotopologues of interest, so they need to be separated and are preferably as low as possible. We use the medium resolution entrance slit and obtained 15,000–17,000 mass resolving power (5%/95% definition), such that $^{2}H^{2}H$ has a plateau wide enough to permit clean measurements on the stacked peak of $^{2}H^{2}H$ and $^{2}H^{1}H_{2}$ (Fig. S12).

Our measurement workflow is similar to previous publications [3,4]. Here we provide a synopsis. Isotopic ratio measurements are made by sample–reference comparisons using the dual inlet system, where the reference is an internal standard gas, 'Nanjing Reference Gas 1'. This reference gas has been determined with $\delta^{2}H_{VSMOW} = -159$ (± 5) ‰, using an external standard (A7 alkane mix). The $\Delta^{2}H^{2}H$ of this reference gas is determined to be 197 ‰ using isotope equilibration experiments, detailed in the next section. The acquisition of the three isotopologues is achieved by jumping the magnet between three positions. Isotopologue abundance is acquired by jumping the magnet in the sequence of $^{1}H^{1}H$ (1.3s)—$^{2}H^{1}H$ (21s)—$^{2}H^{2}H$ (210s), where the time represents integration time for each isotopologue. Each full analysis is composed of one background acquisition and 10 cycles. The background acquisition measures the dark background for the three isotopologues (using the same jumping sequence) with the analyzer gate valve closed. Results from background acquisition are subtracted from actual acquisitions and are also used to help monitor detector instabilities. Each of the 10 analytical cycles begins with a pressure adjustment step for equalizing pressures between both sides, followed by a measurement block of reference–sample–reference–sample–reference. Within this block, each sample or reference is analyzed through the jumping sequence. Isotopologue ratios ($^{2}H^{2}H/^{1}H^{1}H$, $^{2}H^{1}H/^{1}H^{1}H$) for each sample are then bracketed by two references (one before and one after) and are standardized using the reference values.

<u>Precision and reproducibility of analytical results</u>

We generally report internal precision, calculated from standard error of results of acquisition cycles on the Ultra, in this study. The precision for $\Delta^{2}H^{2}H$ is typically within 3–5 ‰ for gas samples with regular $\delta^{2}H_{VSMOW}$ values (Table S2–S3). The errors are larger for the Sichuan Basin gas samples because their depleted $\delta^{2}H_{VSMOW}$ values lead to increased Shot-noise/signal ratios. Our natural gas dataset has a median internal standard error of 15.2 ‰ (Data S1).

In order to test longer term reproducibility, we have conducted recurring measurements of samples from a commercial H_2 tank, 'commercial sample 1' to evaluate consistency of results. This gas sample has a $\delta^{2}H_{VSMOW}$ value 97‰ higher than Nanjing Reference Gas 1. Three measurements of this gas have been made between September 2024 and May 2025.

Their clumped isotope results are statistically consistent (within 2 internal standard errors from each other) and hydrogen isotope results vary slightly larger than 2 internal standard error (Table S3). We also regularly conduct equilibration experiments at 22 °C using H_2 with δ^2H_{VSMOW} = -63 ‰ to monitor instrument stability and analytical accuracy. Between September and November 2024, nine equilibrated gas samples had been prepared and analyzed, yielding consistent results (1 SD = 2.6 ‰; Fig. S13B). This series of results demonstrates the experimental reproducibility of our methods.

Isotope equilibration experiments

Isotope equilibration experiments using metal as catalysts were conducted to establish accuracy of Δ^2H^2H analysis and δ^2H_{VSMOW} analysis in the low δ^2H_{VSMOW} value range. We used Ni/Al_2O_3 (5% weight loading, Sigma-Aldrich) as catalyst for these experiments. We used glass tubes as reactors for these experiments. The glass tubes are about 12 cm long and have a high-vacuum glass valve on the top to allow transfer of gas and seal during experiments. We load the reactor with about 0.2g of Ni/Al_2O_3 catalyst for exchange, 0.6g of molecular sieve (5Å, Sigma-Aldrich) for trapping hydrogen, and some quartz wool to stop the catalyst and molecular sieve from moving up. We evacuate the reactor under 300 °C for 4 hours (or more) to desorb water and other gases, and activate the catalyst by loading 50 μmol H_2 and allow the gas to react under 300 °C for about 1 hour. The reactor is then vacuumed at room temperature. Next, we transfer 50–150 μmol of hydrogen gas into the sample tube immersed in liquid nitrogen. This tube is then placed in a customized tube furnace, made by the Shuangning Yiqi company in Nanjing, China. This furnace allows temperature control within 0.5 ˚C. Sample tubes are heated at different temperatures to allow internal isotopic equilibration; i.e.

$$2\ {}^2H\ {}^1H \rightleftharpoons {}^2H\ {}^2H + {}^1H\ {}^1H.$$

Gas samples are taken from the tube without cooling, and processed (using the method described in the section above) immediately to purify H_2, which is then analyzed for both clumped and hydrogen isotopes. We conducted equilibration experiments at a series of temperatures from 22 to 400 °C. Based on our exploratory tests, 20 hours is long enough for full equilibration at 22 °C, while 1 hour is sufficient for full equilibration at or above 100 °C. The results of 22 °C equilibration experiments (and the theoretical equilibrium Δ^2H^2H value for 22 °C) were used to determine the Δ^2H^2H value of the reference gas and yielded Δ^2H^2H = 197 ‰. This value is used to standardize measurement results to the absolute reference frame.

The experimental results of equilibrating H_2 with regular hydrogen isotope composition (-63 ‰) are consistent with theoretical results for equilibrium fractionation over the temperature range of 22 – 400 °C (Fig. S13A). This confirms that our analytical results are valid across a range of Δ^2H^2H. We also conducted equilibration experiments starting with gas with very low δ^2H (-750 ‰) to examine analytical accuracy across large difference in isotopologue ratios. This is especially important for analyzing natural samples in this study, since their very low δ^2H values differ substantially from our reference gas (δ^2H_{VSMOW} = -159 ‰). Clumped isotopologue abundances for these samples are further much lower following the square law, which is about 7–30 folds less than the reference gas. The initial gas in these experiments with low δ^2H is sourced from natural H_2, separated from natural gas samples. Results indicate that, at the same temperature, equilibrated gas with low δ^2H_{VSMOW} values yield with slightly higher Δ^2H^2H than equilibrated gas with normal δ^2H (Fig. S13A), by 24

‰ on average. Here, we use this offset to correct measured values for samples having low δ^2H_{VSMOW} (< -600 ‰), using this equation:

$$\Delta^2H^2H_{corr} = (\Delta^2H^2H_{meas} + 1)/1.024 - 1$$

, and report the corrected values.

The 24‰ correction applied to low-δ^2H samples is an empirical mass-spectrometric correction, not a thermodynamic correction. Its magnitude is small compared with the large $^2H^2H/^1H^1H$ contrast between low-δ^2H samples and reference gas (about 16 times).

Similarly, such dependence of equilibrated gas clumped isotope values on whole-molecule isotope compositions have been observed in previous analyses in H_2 and CO_2 [4,5]. Possible mechanisms for this type of shift include unaccounted-for detector baseline, isobaric peak contribution and detector non-linearity. As previously described, detector baselines were measured with the gate valve closed, and peak tails from adjacent mass peaks were carefully monitored and corrected. In our collector configuration, $^2H^2H$ was measured on a compact discrete dynode (CDD) ion counter that is known to require careful characterization for dead-time/count-rate effects and non-linear response, whereas $^2H^1H$ and $^1H^1H$ were measured on Faraday cups. These observations suggest that the offset most likely reflects a small non-linearity of the CDD channel under low $^2H^2H$ signal conditions.

Additionally, we conducted a set of equilibration experiments to produce H_2 with a constrained, highly depleted δ^2H_{VSMOW} value, which we used to calibrate the δ^2H_{VSMOW} analysis. This calibration was necessary because some of the analyzed samples have extremely low δ^2H_{VSMOW} values (< -800 ‰), challenging instrument's linearity across a broad range. We conducted equilibration experiments using a similar method from Hochscheid et al.[6]. We carried out these experiments in 12 mL crimp-top glass vials, each loaded with 0.07 g of Ni/Al_2O_3 (5% weight loading, Sigma-Aldrich) as catalyst, and sealed with butyl rubber stoppers. These vials were evacuated until their pressure drops below 0.01 mbar using a needle connected to a rotary pump, then filled with 0.6 bar of H_2. We used syringes to inject 0.5 ml of ultrapure water to each vial, and heat the vials to 50 °C using a heating plate. Stable isotopes of the ultrapure water were analyzed by a liquid water isotope analyzer (TIWA-45-EP by Los Gatos Research) with a result of $\delta^2H_{VSMOW} = -46.1 \pm 0.5$ ‰. The water isotope analyses are calibrated with international standards OH-25, OH-27 and OH-29[7].

Gas samples were then taken from the vial (on the heating plate) using 5 ml gas-tight syringes (Model 1005 SL by Hamilton) and transferred to the vacuum line for purification and then to the Ultra mass spectrometer for analysis. Results indicate that H_2 reached isotopic equilibrium with water after 6 days (Fig. S14). δ^2H_{VSMOW} of the equilibrated H_2 (the last point) is -713.7 ‰, consistent with calculated equilibrium value of -714.9 ‰. This result validates analyses at the low δ^2H_{VSMOW} range, and further prove the linearity for the mass-2 and mass-3 signals acquired by Faraday cups.

<u>Theoretical calculation of kinetic isotope effects</u>

We perform theoretical calculations to obtain the kinetic isotope effects (KIE) for isotopologues of H_2 in elementary reactions of oxidation. We consider the scenarios of H_2 reacting with sulfate and with organic molecules by investigating the elementary reaction of H_2 addition on a few species: SO_4^{2-}, HSO_3^-, toluene radical and methyl radical. SO_4^{2-} and HSO_3^- are selected to represent the oxidation by sulfate minerals. HSO_3^- is chosen instead of SO_3^{2-} because HSO_3^- is a very weak acid (pKa = 7.17 at 25 °C). Methyl and toluene radicals

are selected to represent alkyl and aryl groups, two typical classes of organic species. We do not consider CO_2 as an oxidant because its reaction with H_2 under the temperatures relevant to our study requires Fischer-Tropsch-type synthesis and transition metals as catalysts [8] . This process is known to exchange and equilibrate isotopologues of H_2 rapidly through adsorption [9,10], contrary to our observations.

We follow the principles of transition state theory and statistical mechanical methods proposed by Bigeleisen and Wolfsberg [11] in our KIE calculations. First, we perform a relaxed potential energy surface scan along the presumed reaction coordinate (H–H dissociation) for each reaction to search for the transition state, using the Gaussian 16W software and density functional theory (DFT) at the B3LYP/6-31g(d,p) level. The two reactants of each scan are first optimized separately and then combined into a single system. A relaxed scan is performed using the opt = modredundant keyword, which maps the potential energy surface by varying one internal coordinate while optimizing all others at each step. We use the H-H bond length as internal coordinate for scanning, increasing it incrementally with a step size of 0.1 Å. The resulting energy profile from the scan is examined to identify a peak corresponding to a potential transition state. We perform scans with multiple initial configurations of the two reactants until a distinct energy peak is observed. Results of these scans are presented in Fig. S15.

We use the highest-energy point of the scan as the initial guess to perform geometry optimizations and frequency analysis at the B3LYP/6-311g(d,p) level of theory using the Gaussian 16 software. We verify that the optimized transition state has only one imaginary frequency, and that the vibration mode of this imaginary frequency is on the reaction coordinate (H-H dissociation). We perform an intrinsic reaction coordinate (IRC) scan on the optimized transition states at the B3LYP/6-311g(d,p) level to verify transition states' connectivity. The IRC calculation traces the minimum-energy path from a transition state to reactants and products. IRC results further validate our transition state searches (Fig. S16–S19).

Transition states are presented in Fig. S20 and Data S3. We then conduct frequency analyses for the isotopologues of transition state, where the two hydrogen positions (corresponding to reactant hydrogen) are occupied by: (1) two ^{1}H atoms, (2) one ^{1}H and one ^{2}H in both possible configurations, and (3) two ^{2}H atoms.

Finally, we implement these frequencies to calculate kinetic isotope effects using the formulation by Bigeleisen and Wolfsberg[11]. The ratio of kinetic rate constants between a substituted and a non-substituted isotopologues is calculated as:

$$KIE_{2,1} = \frac{s_2^{\neq} s_1}{s_1^{\neq} s_2}\frac{k_2}{k_1} = \prod_i^{3N-6} \frac{v_{2,i}^{\neq}}{v_{1,i}^{\neq}}\frac{v_{1,i}}{v_{2,i}} \prod_i^{3N-7} \frac{e^{-\frac{u_{2,i}^{\neq}}{2}} * (1-e^{-u_{1,i}^{\neq}})}{e^{-\frac{u_{1,i}^{\neq}}{2}} * (1-e^{-u_{2,i}^{\neq}})} \prod_i^{3N-6} \frac{e^{-\frac{u_{1,i}}{2}} * (1-e^{-u_{2,i}})}{e^{-\frac{u_{2,i}}{2}} * (1-e^{-u_{1,i}})}$$

, and $u_i = \frac{hcv_i}{kT}$.

1 and 2 in the subscripts denote the isotopologues. The superscript ≠ denotes values corresponding to the transition state while absence of a superscript indicates the reactant. The reactant terms refer to the reactant which has isotopic substitution (in our case, H_2). N is the number of atoms; v_i is the harmonic frequency for normal mode i; h is the Planck constant; c is speed of light; k is the Boltzmann constant and T is temperature in Kelvin. For the reactions involving ^{2}H^{1}H, two isotopically distinct transition-state configurations are possible because the ^{2}H and ^{1}H atoms can occupy either of the two non-symmetrical

positions in the transition states (Fig. S20). The total kinetic isotope effect is thus a sum these two configurations: $KIE_{2,1} = (k_{2,t1} + k_{2,t2})/k_1$.

Results covering a temperature range of 0–400 °C are provided in Data S4. We also illustrate the effects of each individual elementary reaction by modeling isotopologue evolution of H_2 through consumption by a single process. Isotopologue ratios of the residual H_2 under consumption with isotopic fractionation are calculated using the Rayleigh equation:

$$R = R_0 * f^{\alpha-1}.$$

R_0 is the initial isotopologue ratio, f is the fraction of H_2 remaining, and α is the fractionation factor. We ran this calculation for diffusion and oxidation by sulfate, sulfite, toluene radical and methyl radical (Fig. S3), using theoretical KIE at 155 °C (the average reservoir temperatures of our samples) for the oxidation reactions.

Calculation of maximum dwell time

As discussed in the main text, isotopic disequilibrium reflects a dynamic state involving recent hydrogen supply or consumption. This inference can be tested by constraining the maximum time that the sampled hydrogen could have remained undisturbed. We define this as the maximum dwell time—the longest possible period during which hydrogen could persist without undergoing exchange or reaction. In practice, we calculate the corresponding exchange times from their current δ^2H_{VSMOW} or Δ^2H^2H values using the isotope exchange model in Pester et al. [12]. We assume a largest possible disequilibrium for the initial isotopic signatures to obtain maximum allowed time. We set initial value for δ^2H_{VSMOW} = -1000 ‰, lowest value allowed by its nomenclature. The nomenclature of Δ^2H^2H does not have upper limits. We therefore set its value to 1000 ‰, higher than all known samples. For each sample, we estimated two independent maximum dwell times derived from δ^2H_{VSMOW} and Δ^2H^2H values, respectively.

Residence time of gas in boreholes

In order to evaluate the potential contribution of anthropogenic H_2 from drill-bit metamorphism, we estimate the typical residence time of gas in boreholes. This is calculated by dividing the typical volume of a drill borehole by the typical gas production rate. Using well-log data, the MX-21 well it has average pipe diameter is 0.2 m and length of 5000 m, yielding a volume of 150 m^3. The typical gas production rate of the MX and GS wells are ~10^6 m^3/day, thus gas residence time is ~10^{-4} day (~10 seconds).

Alternative anthropogenic artifacts

In this section, we discuss the plausibility for a few anthropogenic processes to disturb the observed isotopic signatures. They include isotopic exchange at lower temperature, H_2 generation from corrosive reactions and drilling-induced fluid mixing.

During sampling and storage, isotope exchange could drive H_2 from its initial isotopic composition toward equilibrium values corresponding to lower temperature. However, exchange should drive Δ^2H^2H upwards toward, though not beyond, the value corresponding to 25 °C equilibrium (229 ‰). Instead, we find that 13 out of 30 samples have Δ^2H^2H values exceeding the 25 °C equilibrium value by more than 1 s.e. of analytical error (and 5 by more than 5 s.e.), so re-equilibration at low temperature cannot explain the isotopic properties of collected H_2. Elevated Δ^2H^2H values may be generated by mixing the pristine

H_2 with some fraction of that H_2 that re-equilibrated with H_2O at lower temperature. However, the δ^2H_{VSMOW} values of mixed gas could not be as low as our samples. We thereby conclude that re-equilibration at low temperature is not the dominant control on the isotopic properties of collected H_2. This is expected, as previously reported rates of isotopic exchange between H_2 and water vapor at 25 °C indicate reaction progress of 1.9×10^{-7} day^{-1} [12] — negligible even when integrated over the 1–13 years storage times of our samples.

Hydrogen may also be produced from corrosive reactions between acid and metal materials during gas production or storage. However, previous studies of such reactions suggest $^2\alpha_{H2O–H2}$ between ambient H_2O and produced H_2 is ~1.4 [13], lower than the range of our samples. This finding is also expected, as the interior surfaces of our sample cylinders were passivated with polytetrafluoroethylene (PTFE) before use.

In light of the preceding discussion, the remaining anthropogenic phenomenon that might have caused the isotopic disturbances we observe is activation of chemical reactions with compounds introduced to the gas reservoir via fluid conduits created by drilling. The most plausible candidate for such a process is mixing with oxidant (e.g. sulfate)-rich fluids through drilling. However, this scenario also seems implausible, because the continuous production gas flow should have erased such signatures. We conclude that anthropogenic artifacts cannot explain the isotopic signatures we observed in the central Sichuan Basin gas samples.

Results of theoretical KIE

Theoretical KIE results (Fig. S3) reveal a clear distinction between oxidation by sulfate/bisulfite and by organic radicals. Residual hydrogen oxidized by sulfate and bisulfite exhibit increasing Δ^2H^2H and decreasing δ^2H_{VSMOW}, while that oxidized by toluene and methyl radicals exhibit decreasing Δ^2H^2H and increasing δ^2H_{VSMOW}. Here, we discuss the mechanisms driving this pattern.

In the transition state theory, isotope effects arise from shifts in energy differences between the transition state and the reactant, which can be expressed as partition function ratios. Therefore, the transition state structures of these reactions (Fig. S20) may provide insight into both the direction and magnitude of their kinetic isotope effects (KIEs). Generally, atomic positions involved in stiffer or tighter bonding interactions exhibit higher reduced partition function ratios (RPFRs), reflecting a stronger preference for incorporating heavier isotopes at equilibrium. We found that distances between the attacking H and the target atom are short (<1.0 Å) in transition states of the SO_4^{2-}+H_2 and HSO_3^-+H_2 reactions, but longer (>1.4 Å) in the organic radical reactions (Fig. S20). Also, the vibrational mode corresponding to the stretching of this newly formed bond has a much larger frequency for the SO_4^{2-}/HSO_3^- reactions compared with the organic radical reactions (>3,100 cm^{-1} vs. < 1500 cm^{-1}; Table S4). These observations indicate that the attacking H forms a much stronger bond in the transition states of SO_4^{2-}/HSO_3^- reactions than the organic radical reactions. Thus, their reduced partition function ratios are higher, invoking inversed kinetic isotope effects, while the organic radical reactions show normal isotope effects.

Similarly, the distances between the two hydrogens originally from H_2 are much wider in the transition states of SO_4^{2-}/HSO_3^- reactions than organic molecule reactions. This weaker transition state bond has lower RPFR for the clumped isotopologue, leading to relatively slower $^2H^2H$ consumption. This effect is combined with statistical effect of clumped isotope

nomenclature (i.e. combining two channels of $^2H^1H$ consumption) to produce the pronounced elevation of Δ^2H^2H values in residual H_2.

Isotope effects of diffusion and adsorption

We also calculated the theoretical results of isotopic fractionation in H_2 diffusion. We present results of two scenarios: diffusion in natural gas and in liquid water. In the former scenario, diffusion isotope effects ($\alpha_{A/B} = D_A/D_B$, where D_A and D_B are diffusion coefficients for isotopologue A and B) are approximated with the kinetic theory of gases. It is calculated with $\alpha_{A/B} = \sqrt{m_B(m_A + m_G)/m_A(m_B + m_G)}$, where m_A and m_B are molecular weights of two isotopologues of H_2 and m_G is molecular weight of the background gas through which hydrogen diffuses. Because methane is the dominant component of our gas samples (>90 %), we assign methane as the gas media. Results from this calculation are that $\alpha_{1H2H/1H1H} = 0.8392$ and $\alpha_{2H2H/1H1H} = 0.7458$. In the case of H_2 diffusion in liquid water, we adopt the results of Cao (2022) [14], who robustly calculated isotopologue fractionation factors at 25 °C using molecular dynamics simulations. Their results are $\alpha_{1H2H/1H1H} = 0.9603$ and $\alpha_{2H2H/1H1H} = 0.9289$, much closer to 1 compared with fractionation factors for gas phase diffusion.

Note that diffusion fractionation is an ideal scenario and realistic subsurface transport should be less fractionating because of viscous and molecular flows. We could use methane as an example to demonstrate this. The Knudsen diffusional isotope effect for methane is $\sqrt{M_{16}/M_{17}} = 0.9701$. However, its experimental values obtained from laboratory simulations are generally between 0.995 and 0.999 [15,16]; and its value in the subsurface rocks are estimated to be around 0.999 [17,18].

The 'diffused gas' endmember presented in this study (Fig. 2 and S3) represents the isotopic composition of gas instantaneously released from its source by diffusion. In contrast, the observed gas may have accumulated through progressive diffusion, which would yield isotopic signatures showing reduced fractionation relative to the source.

Diffused H_2 obtained in this study have higher δ^2H_{VSMOW} than those reported in our previous study [1]. This revision reflects methodological updates of using exact gas kinetic equations to calculate dilute H_2 diffusing in mediums of methane or liquid water instead of ideal Knudsen diffusion. The updated calculations therefore provide a more physically realistic estimate of the isotopic effects of H_2 diffusion.

Alternatively, gases that experienced different degrees of diffusive fractionation could mix, potentially producing apparent positive anomalies in Δ^2H^2H. Here we tested the most extreme case of this sort, which is mixing between the instantaneously diffused gas with its source. The calculation results show quite limited positive excursion of Δ^2H^2H (Fig. S3).

Additionally, adsorption may also contribute to isotope fractionation, yet such fractionation has not been investigated to our knowledge. Here we conducted an exploratory experiment to test this effect. We prepared about 0.3 g 5Å molecular sieve (Sigma-Aldrich) and activated it by heating it under vacuum for 4 hours at 300 °C. After it was cooled down to room temperature, we introduced 40 torr of gas into the system and left it for 1 hour. The gas pressure dropped by 10% due to adsorption. We sampled the free gas phase and analyzed it using the protocol described above. The hydrogen and clumped isotope of this free gas was almost not fractionated from the original gas (Table S5). Therefore, we exclude

adsorption as an isotope-fractionating process, although further experiments under varied conditions may be warranted.

Modeling of isotopic evolution

In this section, we detail our calculations of how isotopologue ratios of hydrogen evolve under simultaneous oxidation and exchange. In this mathematical model, these reactions are considered.

$$ {}^{1}H\ {}^{1}H + SO_4^{2-} \rightarrow {}^{1}H\ {}^{1}HO + reduced\ S \quad (1)$$
$$ {}^{2}H\ {}^{1}H + SO_4^{2-} \rightarrow {}^{2}H\ {}^{1}HO + reduced\ S \quad (2)$$
$$ {}^{2}H\ {}^{2}H + SO_4^{2-} \rightarrow {}^{2}H\ {}^{2}HO + reduced\ S \quad (3)$$
$$ {}^{2}H\ {}^{1}H + {}^{1}H\ {}^{1}HO \leftrightarrow {}^{1}H\ {}^{1}H + {}^{2}H\ {}^{1}HO \quad (4)$$
$$ {}^{2}H\ {}^{2}H + {}^{1}H\ {}^{1}HO \leftrightarrow {}^{2}H\ {}^{1}H + {}^{2}H\ {}^{1}HO \quad (5)$$

Reverse reactions of the exchange reaction (4 and 5) are also included in the model. Note that the sulfate reaction mechanisms are simplified and represented as a single, lumped reaction between hydrogen and sulfate, rather than a network of reactions. This approach allows us to calculate the overall isotopic effect using the elementary reaction between SO_4^{2-} and H_2. This simplification is supported by two observations: (1) KIE for HSO_3^- ions is highly similar to that of sulfate (Fig. S3); and (2) sulfur in the oxidation states between sulfite (+4) and sulfide (-2) (e.g. sulfoxylate at +2 oxidation state) is generally not very stable at higher temperatures and would decompose or disproportionate rapidly [19]. Therefore, they would not affect H_2 isotopes directly.

We assume that sulfate is well-supplied, such that the oxidation reactions can be treated as pseudo-first order for hydrogen. I.e., $r_1 = k_{oxi}[\ {}^{1}H\ {}^{1}H]$, where r_1 is the rate of reaction 1. Theoretical KIE for sulfate are implemented in the calculation of reaction 2 and 3: $r_2 = \alpha_{oxi2H1H} k_{oxi}[\ {}^{2}H\ {}^{1}H]$ and $r_3 = \alpha_{oxi2H2H} k_{oxi}[\ {}^{2}H\ {}^{2}H]$. $\alpha_{oxi2H1H}$ and $\alpha_{oxi2H2H}$ are the kinetic isotope fractionation factors of oxidation for $^2H^1H$ and $^2H^2H$, respectively. For the exchange reactions, forward rate of reaction 4 is $r_4 = k_{exc}[\ {}^{2}H\ {}^{1}H][\ {}^{1}H\ {}^{1}HO]$, where k_{exc} is derived from the experimental results (exchanging with saturated vapor) by Pester et al. [12]. For the forward reactions of reaction 5, $r_5 = 2k_{exc}[\ {}^{2}H\ {}^{2}H][\ {}^{1}H\ {}^{1}HO]$. A factor of 2 here is included because of the symmetry number ratio of reaction 5 is twice that of reaction 4 (i.e. 2 vs. 1). Readers can refer to Goldman et al. [20] for proper definition of the kinetic rate constants of isotopologue reactions. The kinetic rate constants of reverse reactions of reaction 4 and 5 are calculated as kinetic rate constants of their forward counterparts divided by their equilibrium constants. Equilibrium constants for these reactions are based on data from the experimental calibration for H_2O–H_2 by Hochscheid et al. [6] and *ab initio* theoretical results for clumped isotopes of H_2 by Korol et al. [21].

We consider two scenarios: a closed system and an open system. In the closed system, the concentrations of hydrogen isotopologues are controlled entirely by oxidation and exchange reactions (reaction 1–5). In the open system, we additionally incorporate input and output fluxes of H_2 to emulate a constant source and a constant physical loss process. The input flux is assumed to be constant, while the output flux is set to be proportional to the amount of hydrogen present in the system. We then derive ordinary differential equations for both systems and solve them using the ode23s method included by the MATLAB software, which is a low order method based on a modified Rosenbrock formula of order 2. Absolute tolerance is set to 1e-15.

Initial δ^2H_{VSMOW} of water is -50 ‰. Initial abundance of water is treated as saturated vapor (0.5 Mpa at 155 °C). Initial condition for H_2 is that $[{}^2H\ {}^1H]/[{}^1H\ {}^1H]$ and $[{}^2H\ {}^2H]/[{}^1H\ {}^1H]$ are in equilibrium both with water and internally corresponding to temperature of the basement rock, estimated to be 178 °C. We assume isotopic equilibria condition for initial gas because hydrothermal-derived hydrogen is usually under isotopic equilibrium and isotopic exchange reaction at 178 °C can happen very rapidly [12]. We estimated the temperature of the basement rock using its typical starting depth (6 km, Fig. 1) and average geothermal gradient for the wells of our sample set, 0.029 °C/m (Data S1). We select a range of k_{oxi} values to present model outputs.

We assigned multiple parameters for the model, including reaction temperature, initial concentrations of reactants and input and output fluxes. We conduct series of tests to evaluate the model's sensitivity with varying parameters. We test a range of input and output flux strengths in the open-system model (Fig. S5). The results show a noticeable shift in isotopic composition with varying output strength, whereas input flux variations have relatively minor effects. In the closed-system model, varying the initial H_2 partial pressure produces only minimal changes in the results (Fig. S6). We also vary the temperature between 90 and 175 °C for both closed-system and open-system models, and find that lower reaction temperatures can produce more pronounced depletion in δ^2H_{VSMOW} values (Fig. S7 and S8). This temperature effect improves the alignment between model outputs and some of the observed sample data, especially for the open-system scenario. However, these lower reaction temperatures are not realistic for reservoirs under higher temperatures. Overall, the results suggest that the modeled outputs are relatively insensitive to a broad range of parameter variations, indicating consistent outcome.

For the presentation of model results in main-text Fig. 4, we set temperature for the oxidation and equilibration processes to be 155 °C (the average reservoir temperatures of our samples). For the closed-system model, we initiated H_2 with a partial pressure of 20 MPa; for the open-system model, we set initial H_2 partial pressure to 0.5 MPa, H_2 input flux to 0.1 $MPa \cdot yr^{-1}$, and output flux rate to 10^{-4} yr^{-1}.

Constraining oxidation kinetics with Monte-Carlo analyses

We constrain the oxidation rates of each individual sample using the Monte-Carlo method. This is done by running the closed-system oxidation-exchange model with randomized parameters and collecting parameter sets that produce results consistent with the sample data. For each Monte Carlo simulation, $log(k_{oxi}(yr^{-1}))$ is drawn from a uniform distribution of [-5, 1].

The Monte Carlo simulation is executed to generate 50,000 sets of output from the oxidation-exchange model. We calculate the normalized distance between the data points in the output of each single simulation and the samples data (for all 30 samples), using this equation:

$$dist\_n = \frac{\left(\delta^2H_{model} - \delta^2H_{sample}\right)^2}{(\sigma\delta^2H)^2} + \frac{\left(\Delta^2H^2H_{model} - \Delta^2H^2H_{sample}\right)^2}{(\sigma\Delta^2H^2H)^2}$$

$\sigma\delta^2H$ and $\sigma\Delta^2H^2H$ are analytical errors for δ^2H and Δ^2H^2H, respectively. If the minimum $dist\_n$ of a model output is smaller than 2, this set of parameters used in the model is considered a match with this sample. 28 out of 30 samples yield matching results in this analysis. For each sample, we collect $\log(k_{oxi})$ values from the matching Monte-Carlo runs and calculate its mean and standard deviation (Fig. S21). For the gas samples, k_{oxi} values

have a median of 1.1 yr^{-1} and range from 0.015 to 3.5 yr^{-1} (Data S5). Mean value for k_{oxi} is 1.2 yr^{-1}. We conduct bootstrap analyses using 1,000 bootstrap samples to obtain its standard deviation of the mean to be 0.14 yr^{-1}.

We further estimate total oxidation flux for the studied area using this median value. This could be done by multiplying the total natural gas reserve with the average reconstructed original H_2 abundance. Total natural gas reserve in the central Sichuan Basin area has been estimated to be 5×10^{12} m^3 according to Zhang 2022 [22]. Note that our sample set is a high-H_2 subset selected from the full sample set with 58 samples (due to the sample size requirement for clumped isotope analysis), where the rest of the samples have been reported by Wang et al. [1]. To avoid selection bias, we include the H_2 abundances of the full set samples for estimation of total H_2 amounts. These calculations yield mean current H_2 abundance of 0.30 %. We conduct bootstrap analyses using 1,000 bootstrap samples to obtain the standard deviation of the mean current abundance to be 4.1×10^{-4}. By multiplying the H_2 abundance with total gas reserve, the total current H_2 is estimated to be $1.3 \pm 0.4 \times 10^6$ tons (95% CI). Finally, this value is multiplied with mean k_{oxi} of 1.2 ± 0.1 yr^{-1} to obtain hydrogen loss rate of $1.6 \pm 0.7 \times 10^6$ tons/yr (95% CI).

References

1. Wang X, He K, Yang C *et al.* Formation mechanism of deep natural hydrogen in the Sichuan Basin. *Science China Earth Sciences 2025 68:2* 2025;**68**(2):581–97. https://doi.org/10.1007/S11430-024-1463-1.
2. Zou C, Du J, Xu C *et al.* Formation, distribution, resource potential, and discovery of Sinian–Cambrian giant gas field, Sichuan Basin, SW China. *Petroleum Exploration and Development* 2014;**41**(3):306–25. https://doi.org/10.1016/S1876-3804(14)60036-7.
3. Popa ME, Paul D, Janssen C *et al.* H 2 clumped isotope measurements at natural isotopic abundances. *Rapid Communications in Mass Spectrometry* 2019;**33**(3):239–51. https://doi.org/10.1002/rcm.8323.
4. Mangenot X, Xie H, Crémière A *et al.* 2H-2H clumping in molecular hydrogen method and preliminary results. *Chem Geol* 2023;**621**:121278. https://doi.org/10.1016/J.CHEMGEO.2022.121278.
5. Dennis KJ, Affek HP, Passey BH *et al.* Defining an absolute reference frame for 'clumped' isotope studies of CO2. *Geochim Cosmochim Acta* 2011;**75**(22):7117–31. https://doi.org/10.1016/J.GCA.2011.09.025.
6. Hochscheid F, Turner AC, Lotem N *et al.* Experimental determination of hydrogen isotopic equilibrium in the system H2O(l)-H2(g) from 3 to 90 °C. *Geochim Cosmochim Acta* 2025;**394**:368–82. https://doi.org/10.1016/J.GCA.2025.02.029.
7. Wassenaar L, Terzer-Wassmuth S, Douence C. Progress and challenges in dual- and triple-isotope (δ18O, δ2H, Δ17O) analyses of environmental waters: An international assessment of laboratory performance. *Rapid Communications in Mass Spectrometry* 2021;**35**(24):e9193. https://doi.org/10.1002/RCM.9193.
8. McCollom TM, Seewald JS. Abiotic synthesis of organic compounds in deep-sea hydrothermal environments. *Chem Rev* 2007;**107**(2):382–401. https://doi.org/10.1021/CR0503660.

9. OBrien CP, Miller JB, Morreale BD *et al.* The kinetics of H 2-D 2 exchange over Pd, Cu, and PdCu surfaces. *Journal of Physical Chemistry C* 2011;**115**(49):24221–30. https://doi.org/10.1021/JP2076885/ASSET/IMAGES/JP-2011-076885_M019.GIF.

10. Christmann K. Interaction of hydrogen with solid surfaces. *Surf Sci Rep* 1988;**9**(1–3):1–163. https://doi.org/10.1016/0167-5729(88)90009-X.

11. Bigeleisen J, Wolfsberg M. *Theoretical and Experimental Aspects of Isotope Effects in Chemical Kinetics*. n.p.: John Wiley & Sons, Ltd, 1957, 15–76. https://doi.org/10.1002/9780470143476.ch2.

12. Pester NJ, Conrad ME, Knauss KG *et al.* Kinetics of D/H isotope fractionation between molecular hydrogen and water. *Geochim Cosmochim Acta* 2018;**242**:191–212. https://doi.org/10.1016/J.GCA.2018.09.015.

13. Feldbusch E, Wiersberg T, Zimmer M *et al.* Origin of gases from the geothermal reservoir Groß Schönebeck (North German Basin). *Geothermics* 2018;**71**:357–68. https://doi.org/10.1016/J.GEOTHERMICS.2017.09.007.

14. Cao X. Diffusional isotope fractionation of singly and doubly substituted isotopologues of H2, N2 and O2 during air-water gas transfer. *Geochim Cosmochim Acta* 2022;**332**:78–87. https://doi.org/10.1016/J.GCA.2022.06.022.

15. Zhang T, Krooss BM. Experimental investigation on the carbon isotope fractionation of methane during gas migration by diffusion through sedimentary rocks at elevated temperature and pressure. *Geochim Cosmochim Acta* 2001;**65**(16):2723–42. https://doi.org/10.1016/S0016-7037(01)00601-9.

16. Schloemer S, Krooss BM. Molecular transport of methane, ethane and nitrogen and the influence of diffusion on the chemical and isotopic composition of natural gas accumulations. *Geofluids* 2004;**4**(1):81–108. https://doi.org/10.1111/J.1468-8123.2004.00076.X.

17. Li W, Lu S, Li J *et al.* Geochemical modeling of carbon isotope fractionation during methane transport in tight sedimentary rocks. *Chem Geol* 2021;**566**:120033. https://doi.org/10.1016/J.CHEMGEO.2020.120033.

18. Xia X, Tang Y. Isotope fractionation of methane during natural gas flow with coupled diffusion and adsorption/desorption. *Geochim Cosmochim Acta* 2012;**77**:489–503. https://doi.org/10.1016/J.GCA.2011.10.014.

19. Tossell JA. Theoretical studies on possible sulfur oxides with +2 oxidation states in aqueous solution. *Chem Geol* 1997;**141**(1–2):93–103. https://doi.org/10.1016/S0009-2541(97)00065-X.

20. Goldman MJ, Ono S, Green WH. Correct Symmetry Treatment for X + X Reactions Prevents Large Errors in Predicted Isotope Enrichment. *Journal of Physical Chemistry A* 2019;**123**(12):2320–4. https://doi.org/10.1021/ACS.JPCA.8B09024/ASSET/IMAGES/MEDIUM/JP-2018-09024P_M028.GIF.

21. Korol R, Turner AC, Nandi A *et al.* Stable isotope equilibria in the dihydrogen-water-methane-ethane-propane system. Part 1: Path-integral calculations with CCSD(T) quality potentials. *Geochim Cosmochim Acta* 2025;**396**:71–90. https://doi.org/10.1016/J.GCA.2025.02.028.

22. Zhang D. Development prospect of natural gas industry in the Sichuan Basin in the next decade. *Natural Gas Industry B* 2022;**9**(2):119–31. https://doi.org/10.1016/J.NGIB.2021.08.025.

23. Turner AC, Korol R, Eldridge DL *et al.* Experimental and theoretical determinations of hydrogen isotopic equilibrium in the system CH4H2H2O from 3 to 200 °C. *Geochim Cosmochim Acta* 2021;**314**:223–69. https://doi.org/10.1016/J.GCA.2021.04.026.
24. Vacquand C, Deville E, Beaumont V *et al.* Reduced gas seepages in ophiolitic complexes: Evidences for multiple origins of the H2-CH4-N2 gas mixtures. *Geochim Cosmochim Acta* 2018;**223**:437–61. https://doi.org/10.1016/J.GCA.2017.12.018.
25. Maiga O, Deville E, Laval J *et al.* Characterization of the spontaneously recharging natural hydrogen reservoirs of Bourakebougou in Mali. *Scientific Reports 2023 13:1* 2023;**13**(1):1–13. https://doi.org/10.1038/s41598-023-38977-y.
26. Truche L, Donzé FV, Goskolli E *et al.* A deep reservoir for hydrogen drives intense degassing in the Bulqizë ophiolite. *Science (1979)* 2024;**383**(6683):618–21. https://doi.org/10.1126/SCIENCE.ADK9099/SUPPL_FILE/SCIENCE.ADK9099_MOVIE_S1.ZIP.
27. Jenden PD, Drazan DJ, Kaplan IR. Mixing of thermogenic natural gases in northern Appalachian Basin. *American Association of Petroleum Geologists Bulletin* 1993;**77**(6):980–98. https://doi.org/10.1306/bdff8dbc-1718-11d7-8645000102c1865d.
28. Guélard J, Beaumont V, Rouchon V *et al.* Natural H2 in Kansas: Deep or shallow origin? *Geochemistry, Geophysics, Geosystems* 2017;**18**(5):1841–65. https://doi.org/10.1002/2016GC006544.
29. Boreham CJ, Sohn JH, Cox N *et al.* Hydrogen and hydrocarbons associated with the Neoarchean Frog's Leg Gold Camp, Yilgarn Craton, Western Australia. *Chem Geol* 2021;**575**:120098. https://doi.org/10.1016/J.CHEMGEO.2021.120098.
30. Templeton AS, Ellison ET, Kelemen PB *et al.* Low-temperature hydrogen production and consumption in partially-hydrated peridotites in Oman: implications for stimulated geological hydrogen production. *Frontiers in Geochemistry* 2024;**2**:1366268. https://doi.org/10.3389/FGEOC.2024.1366268.

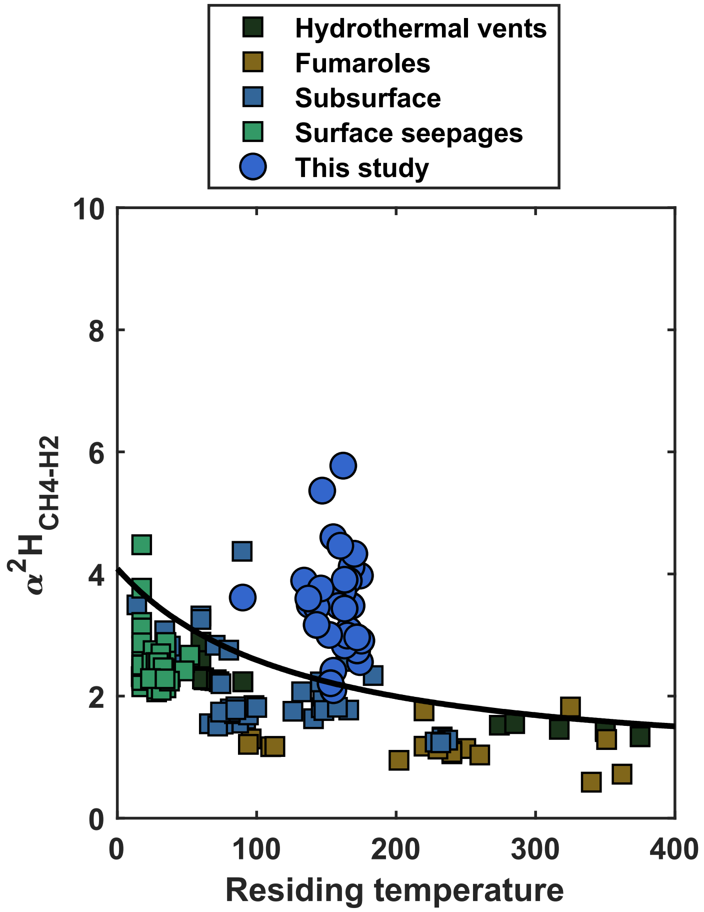

Fig. S1. Methane–H_2 hydrogen isotope fractionation of the central Sichuan Basin natural gases (this study) and various other origins from the literature (Data S2). The surface seepage type includes gases found in springs, seeps and outcrop rock samples. The residing temperature refers to the temperature at which the gas is found at the time of sampling, determined either through direct measurement or inferred from geothermal gradients (specified in Data S2). Black curve is the thermodynamic equilibrium fractionation from Turner et al. [23].

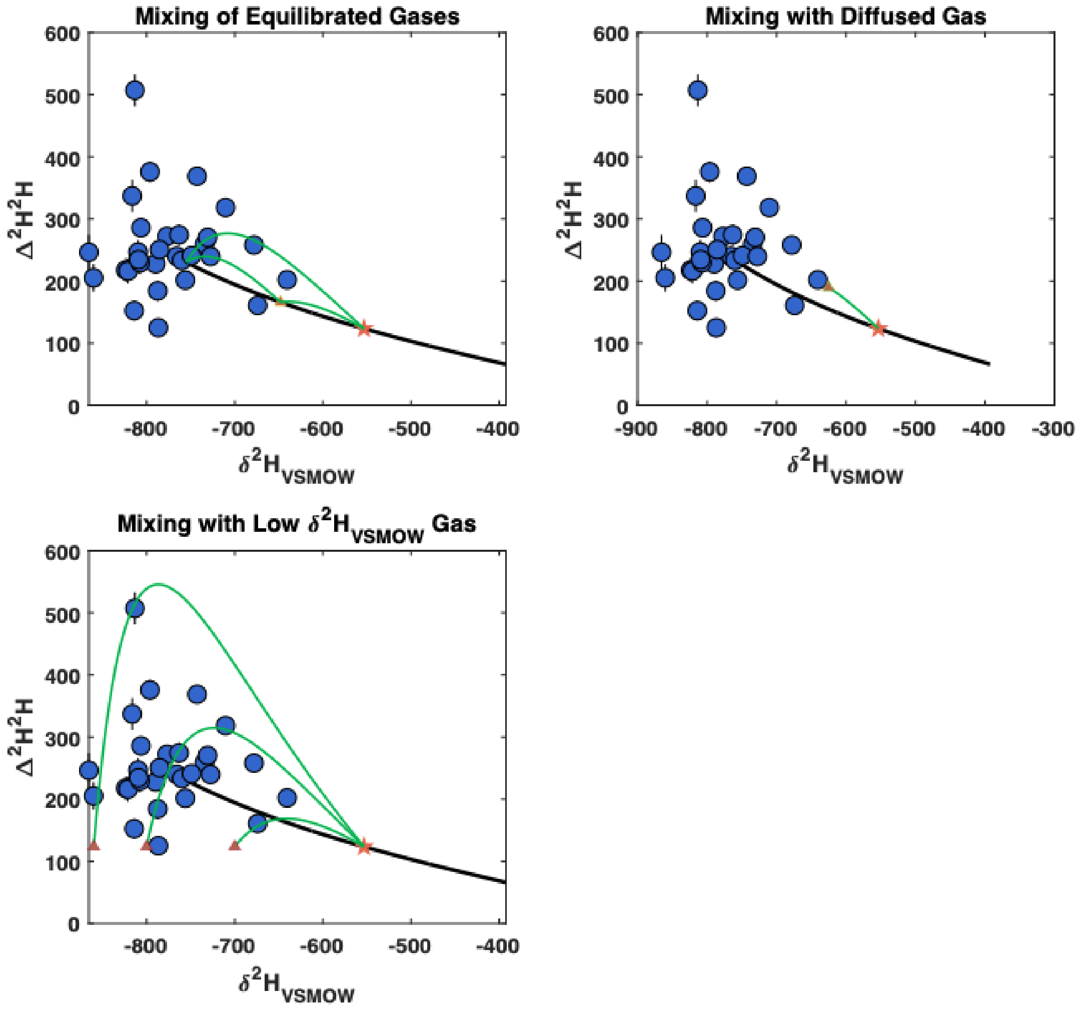

Fig. S2. Evaluation of the mixing effect. We consider three scenarios: mixing with low temperature equilibrated gases; mixing with diffused gas; and mixing with hypothetical low δ^2H_{VSMOW} gases. For the low temperature equilibrated gases, we used equilibration temperatures of 25 and 100 °C. For the diffused gas, theoretical results of H_2 diffusion in CH_4 is used. For the hypothetical low δ^2H_{VSMOW} gases, we used these three endmember scenarios: δ^2H_{VSMOW} = -860, -800 and -700 ‰.

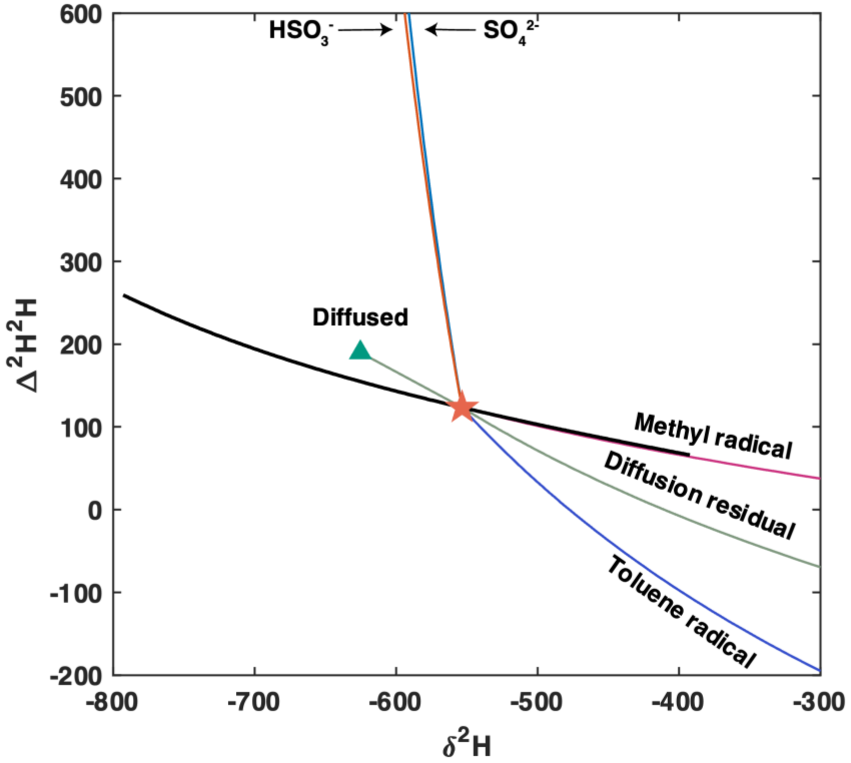

Fig. S3. Theoretically calculated clumped and hydrogen isotope evolution in a variety of processes. The ‘diffused’ and ‘diffusion residual’ represent theoretical results of H_2 diffusion in CH_4. The other results represent isotopic composition of residual H_2 being oxidized by a variety of oxidants.

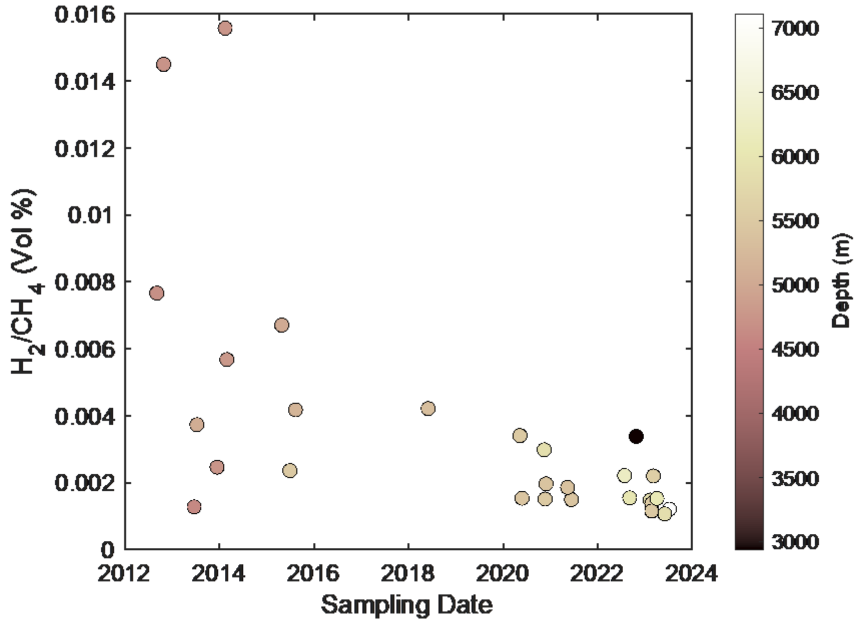

Fig. S4. Hydrogen content of gas samples in this study, vs. their sampling dates. Markers are color-coded with sampling depth.

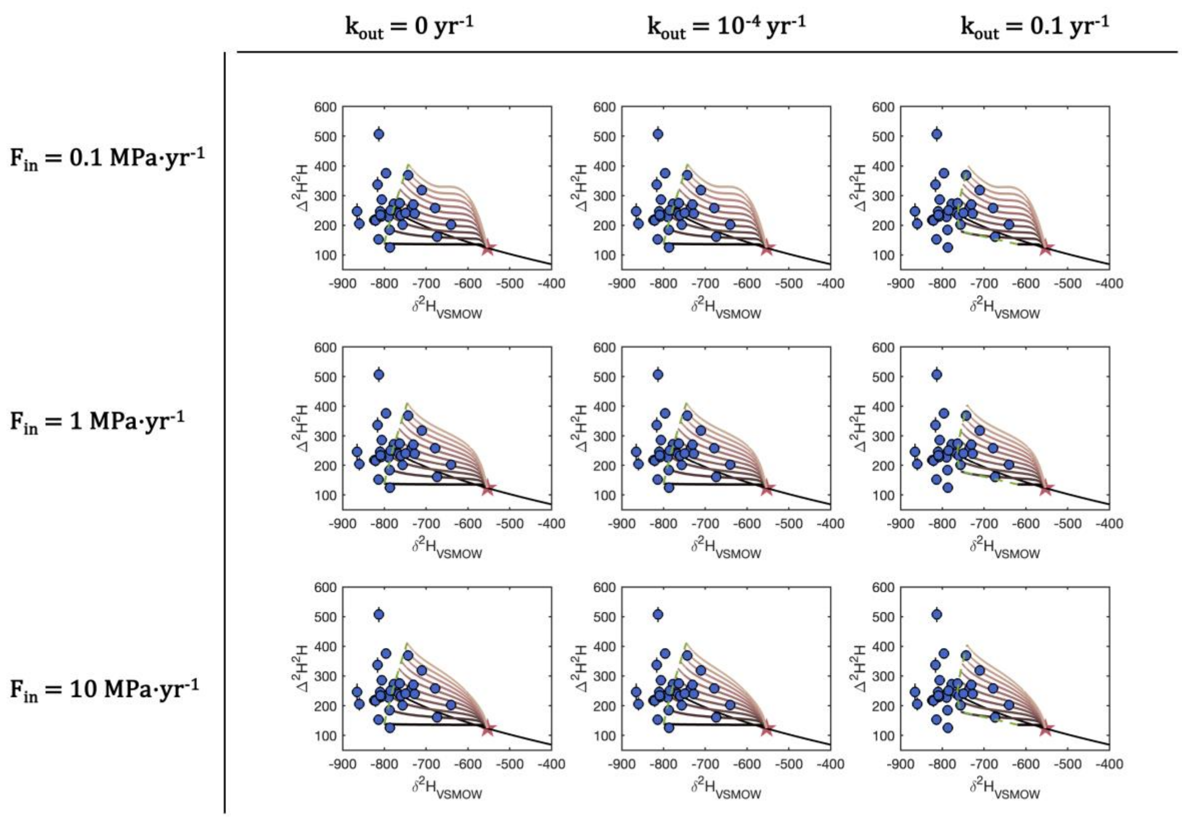

Fig. S5. Sensitivity analysis of input and output H_2 flux parameters in the open-system isotope-evolution model. These simulations are run with initial H_2 partial pressure of 5 MPa and reaction temperature of 155 °C.

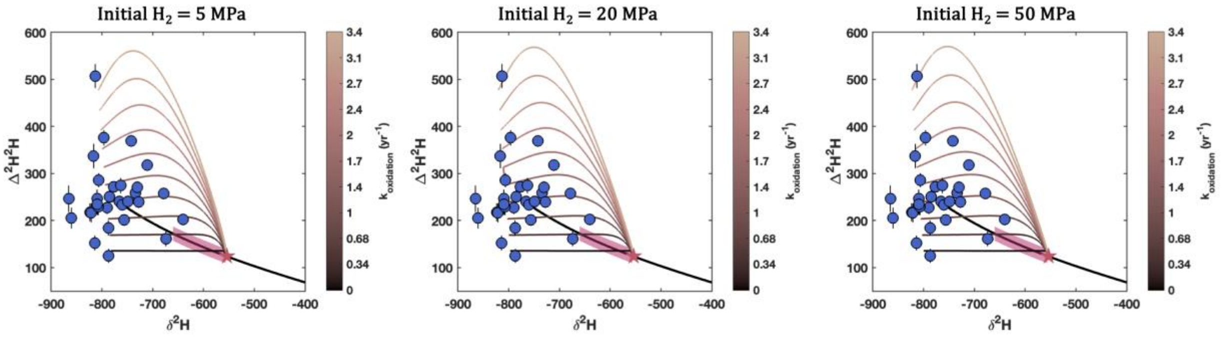

Fig. S6. Sensitivity analysis of initial H_2 partial pressure in the closed-system isotope-evolution model. These simulations are run with reaction temperature of 155 °C.

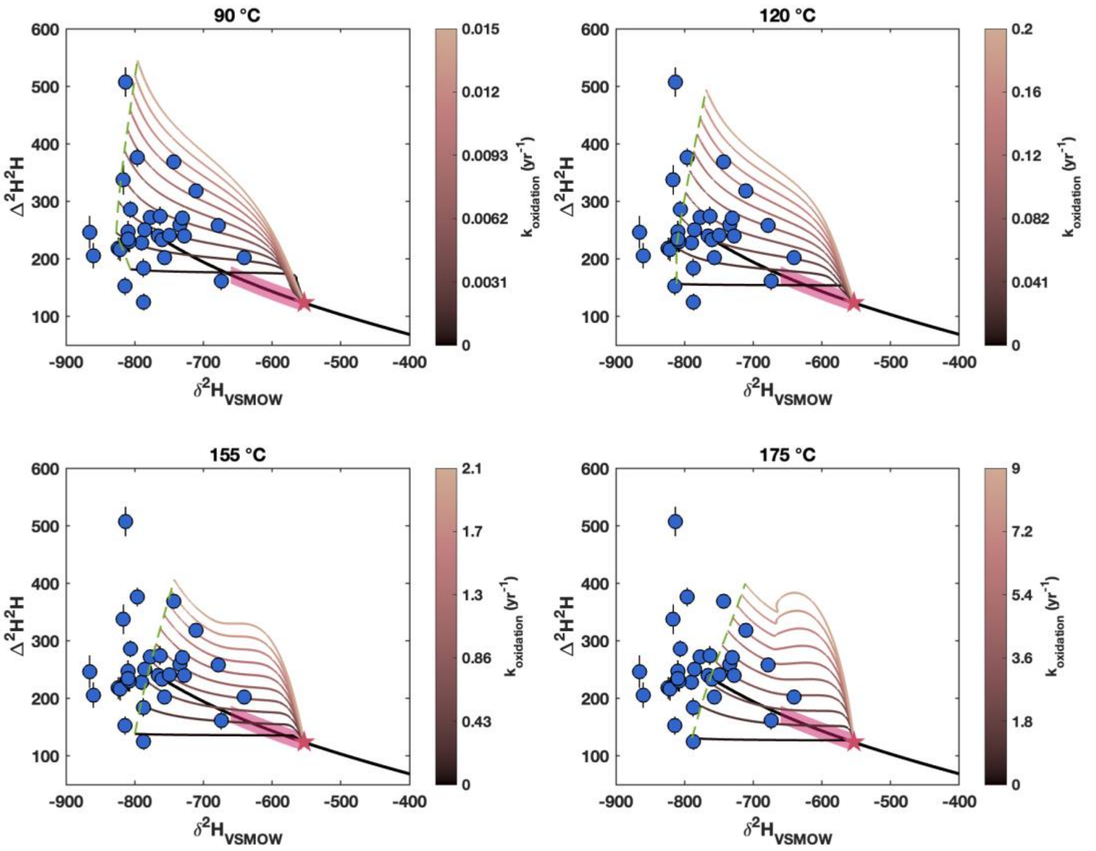

Fig. S7. Sensitivity analysis of reaction temperature in the open-system isotope-evolution model. These simulations assume initial H_2 partial pressure of 5 MPa, input flux of 1 MPa·yr^{-1}, and output rate of 10^{-4} yr^{-1}.

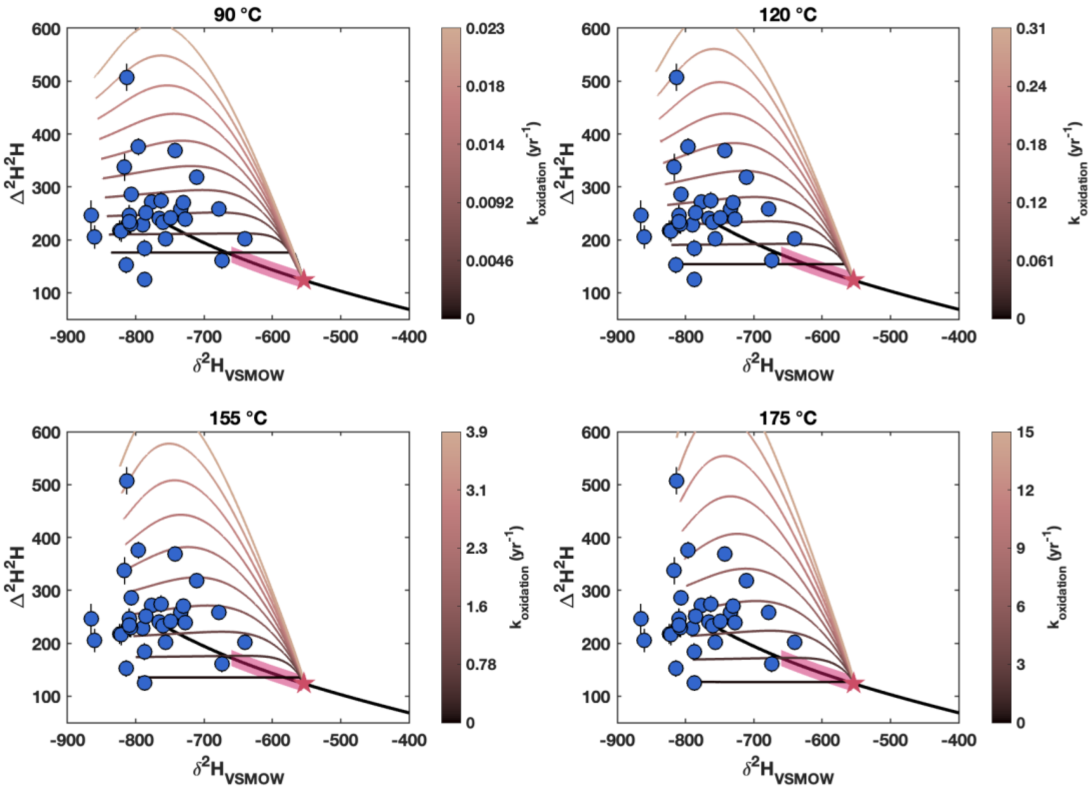

Fig. S8. Sensitivity analysis of reaction temperature in the closed-system isotope-evolution model. These simulations are run with initial H_2 partial pressure of 20 MPa.

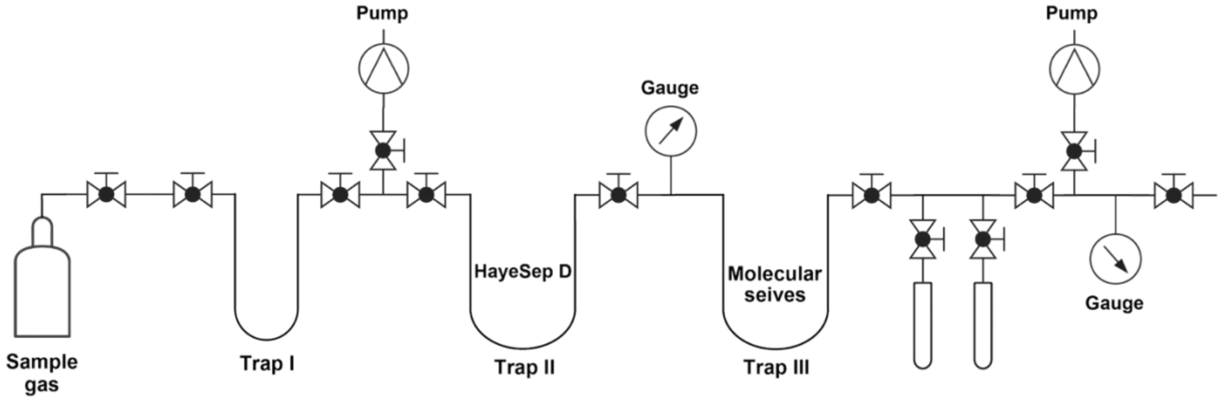

Fig. S9. Schematics of the gas vacuum line used for natural hydrogen purification.

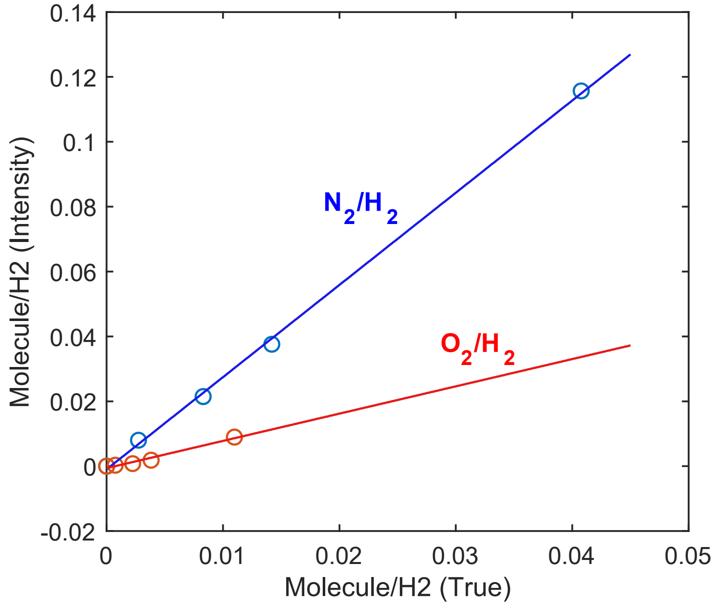

Fig. S10. Responses of mixing N_2 and O_2 with H_2 on the Ultra mass spectrometer, for evaluating contaminant abundances.

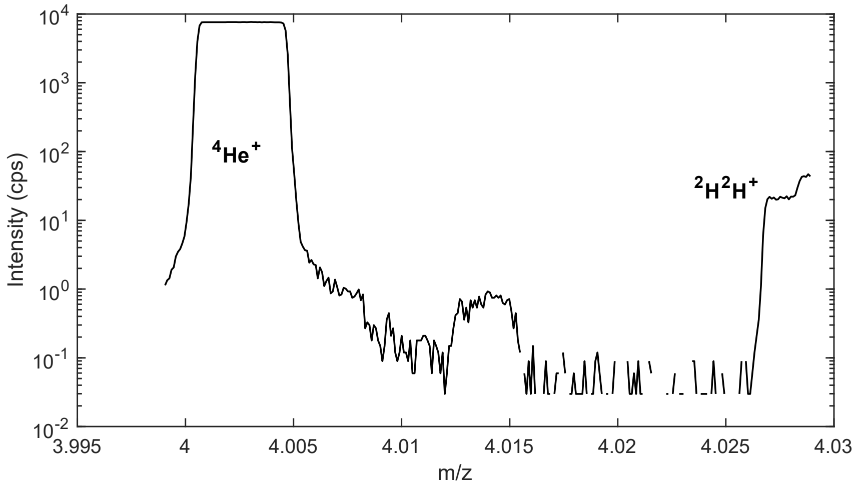

Fig. S11. Mass spectrum of the m/z 4 region with y-axis on the logarithmic scale. This spectrum illustrates ^{4}He's potential influence on the $^{2}H^{2}H$ analysis.

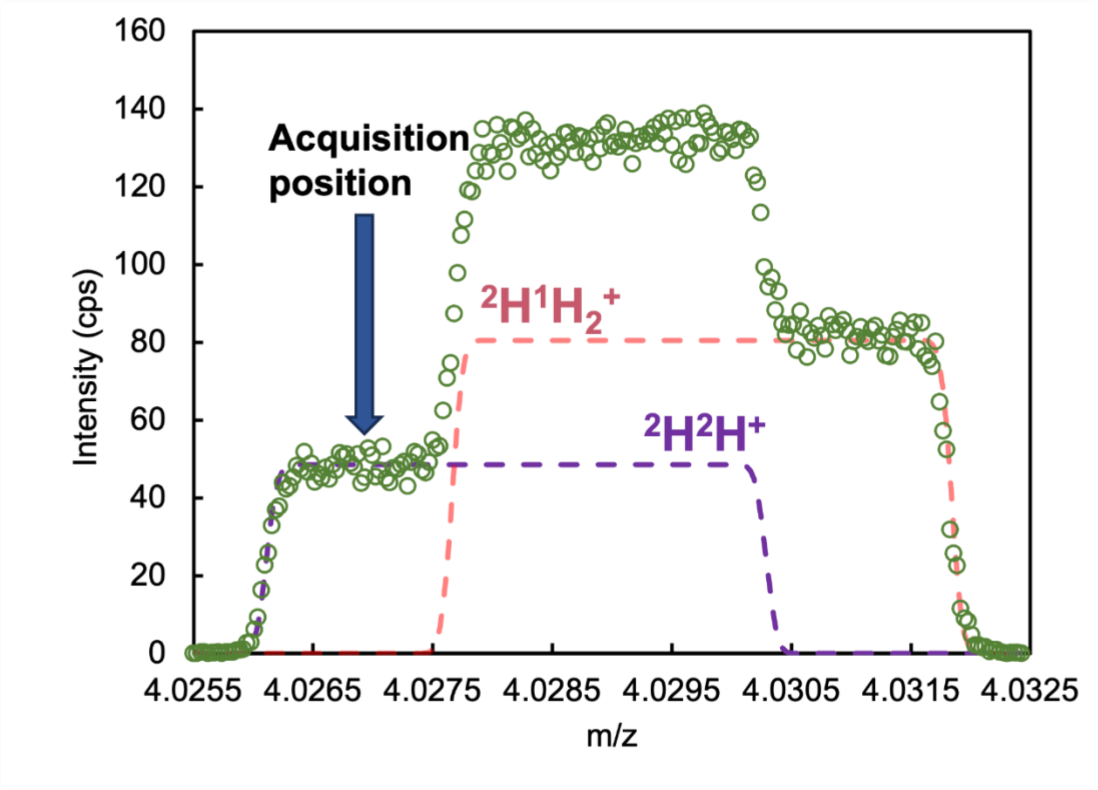

Fig. S12. Mass spectrum of the m/z 4 peaks of H_2 acquired by the Ultra mass spectrometer.

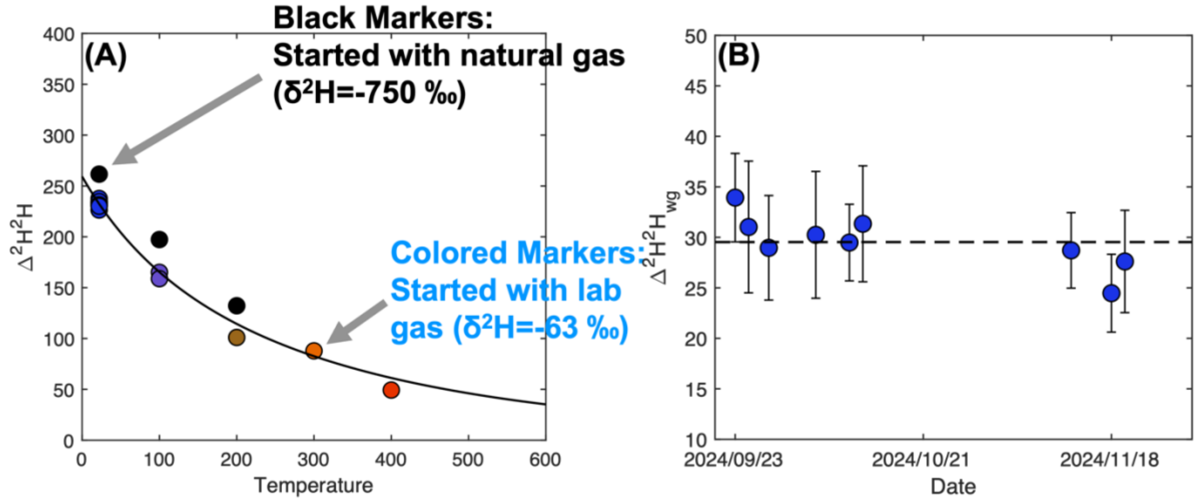

Fig. S13. Results of H_2 clumped isotope equilibration experiments. (A). Clumped isotope values of experimentally equilibrated gas at various temperatures in °C. (B). Clumped isotope values (relative to the working gas reference frame) of experimentally equilibrated gas at 22 °C.

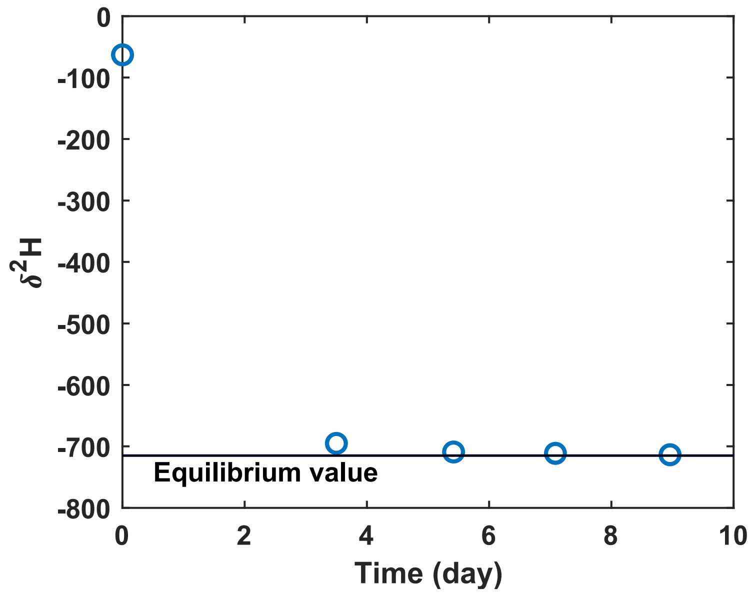

Fig. S14. Results of a H_2O–H_2 exchange experiment at 50 °C. Analytical errors are smaller than the markers.

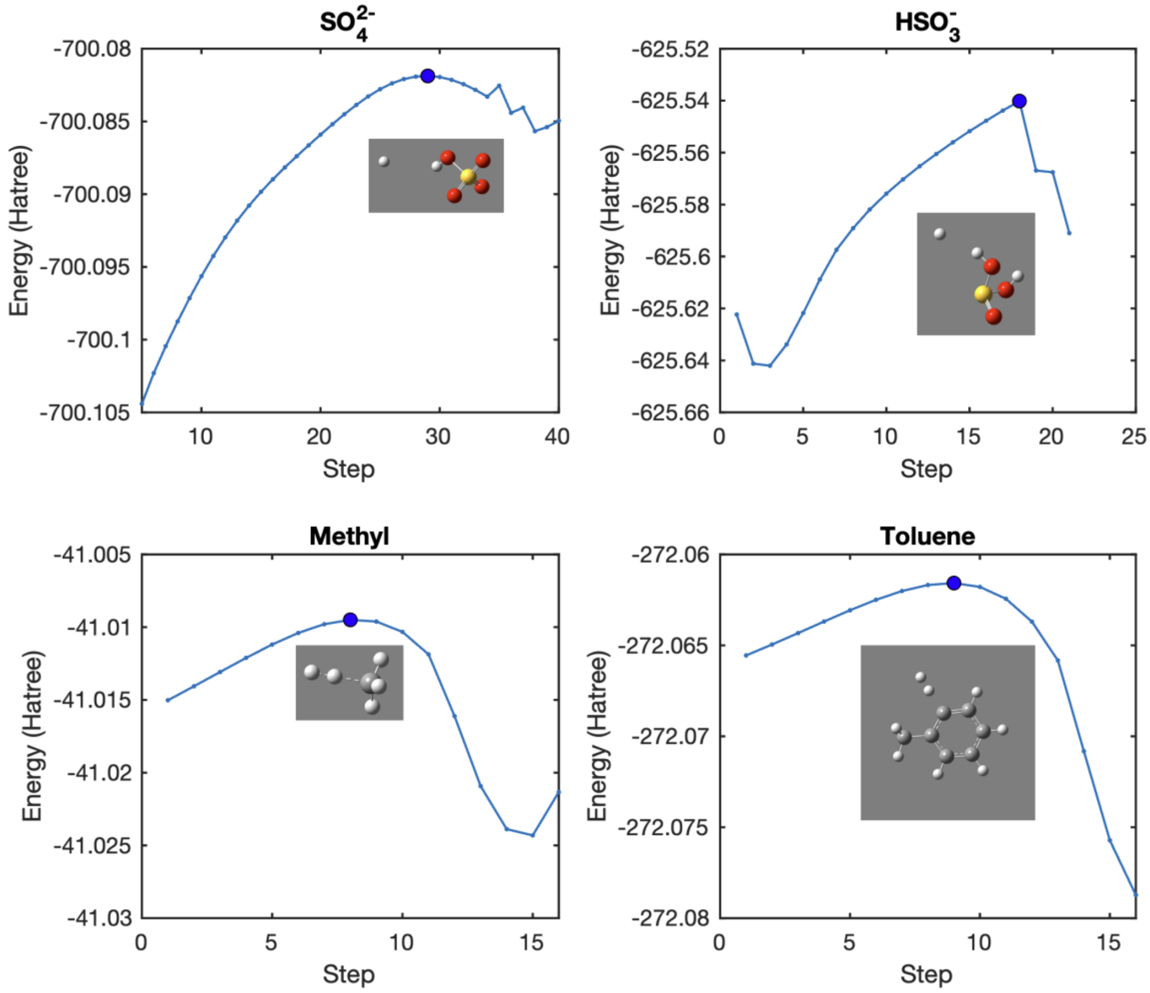

Fig. S15. Potential energy surface scans along the presumed reaction coordinate for oxidation reactions. These scans allow the coordinates not being scanned to be optimized (a.k.a., relaxed scan). Molecular image shows configuration at the peak energy.

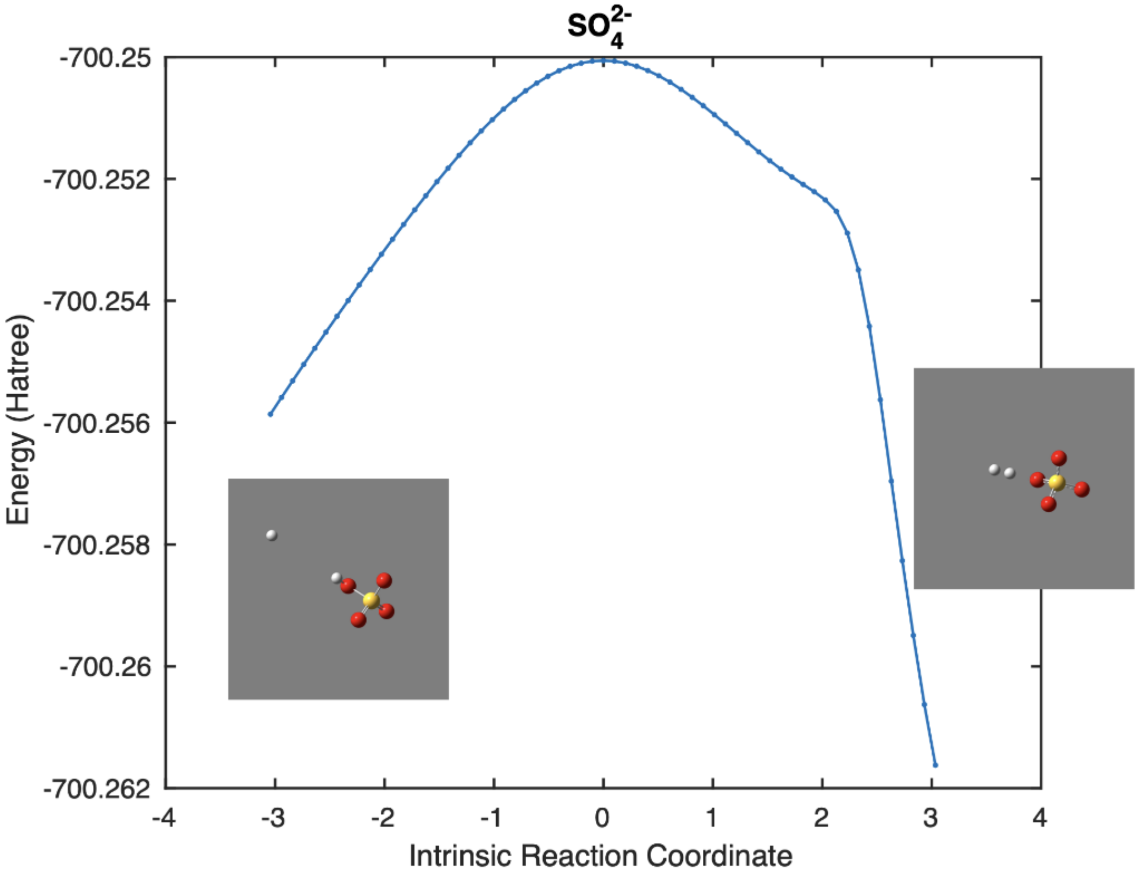

Fig. S16. Intrinsic reaction coordinate scan for SO_4^{2-}+H_2 reaction. Molecular images of the terminal points are displayed.

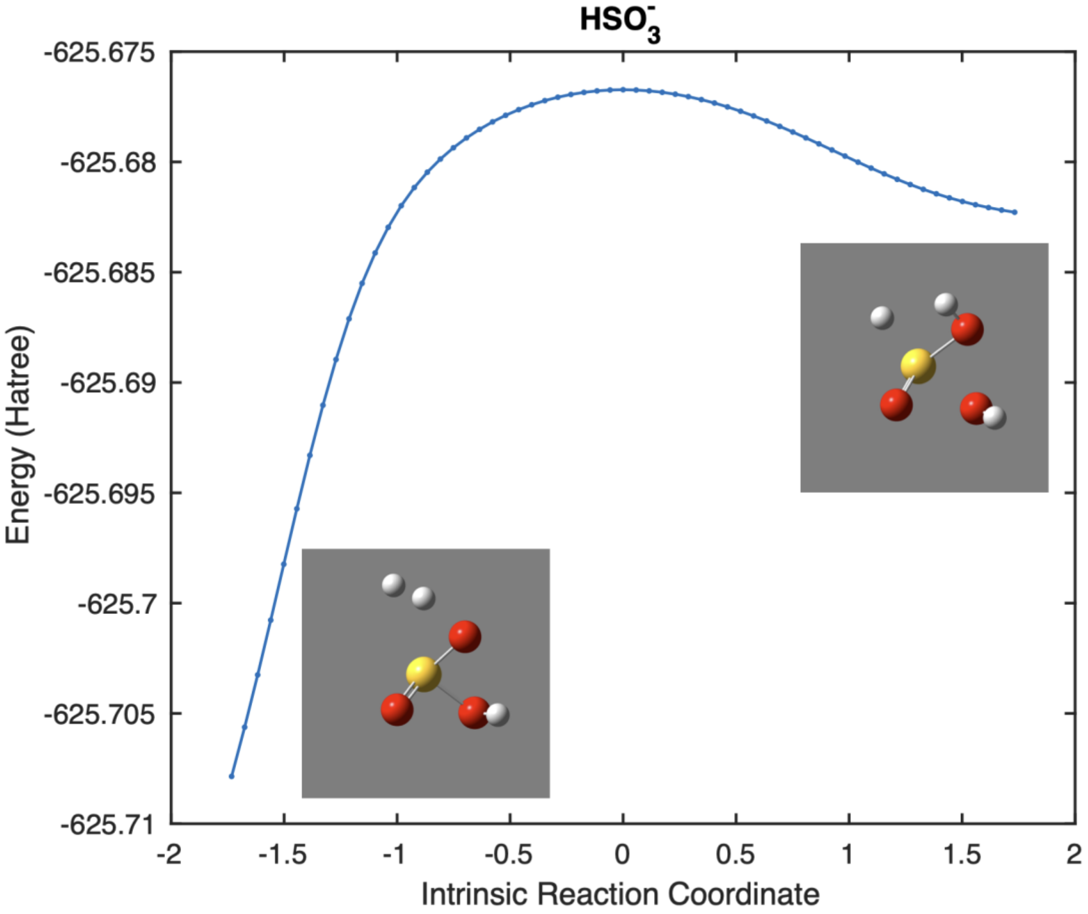

Fig. S17. Intrinsic reaction coordinate scan for HSO_3^-+H_2 reaction. Molecular images of the terminal points are displayed.

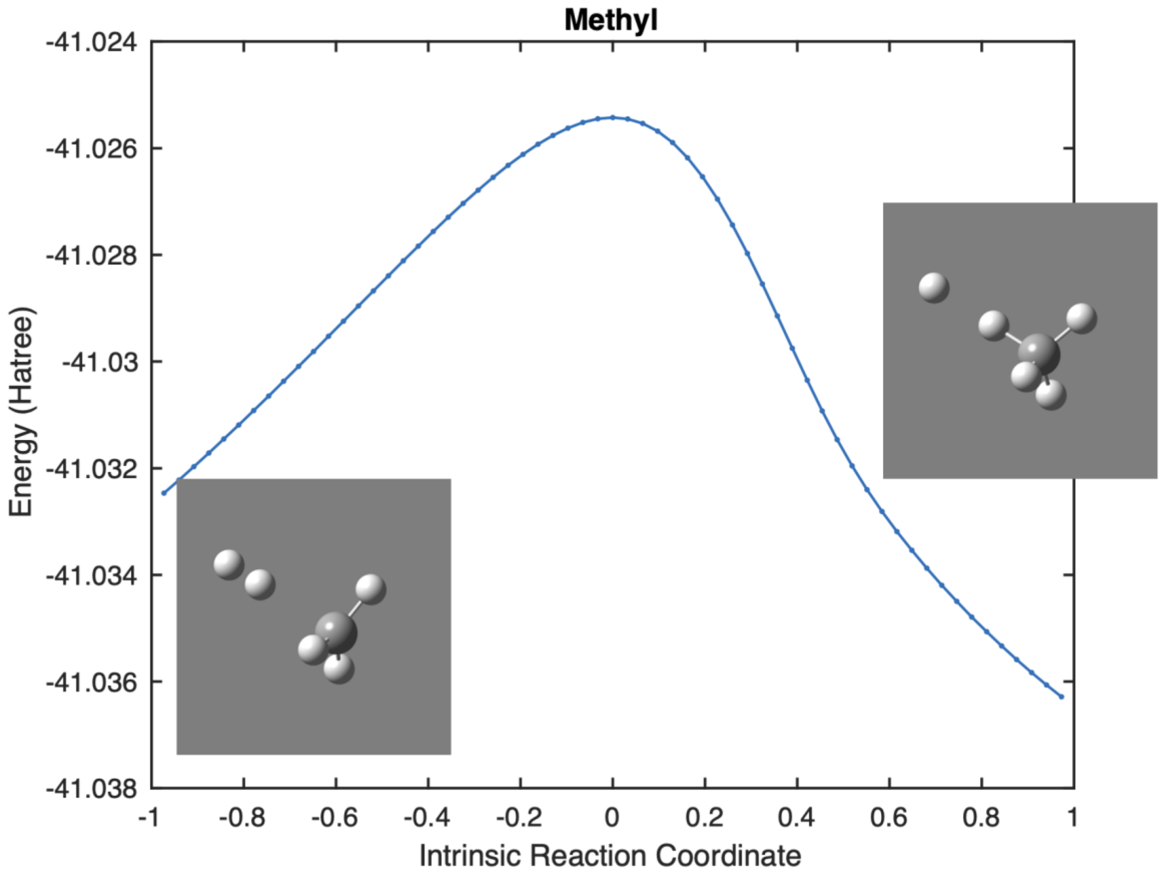

Fig. S18. Intrinsic reaction coordinate scan for CH_3+H_2 reaction. Molecular images of the terminal points are displayed.

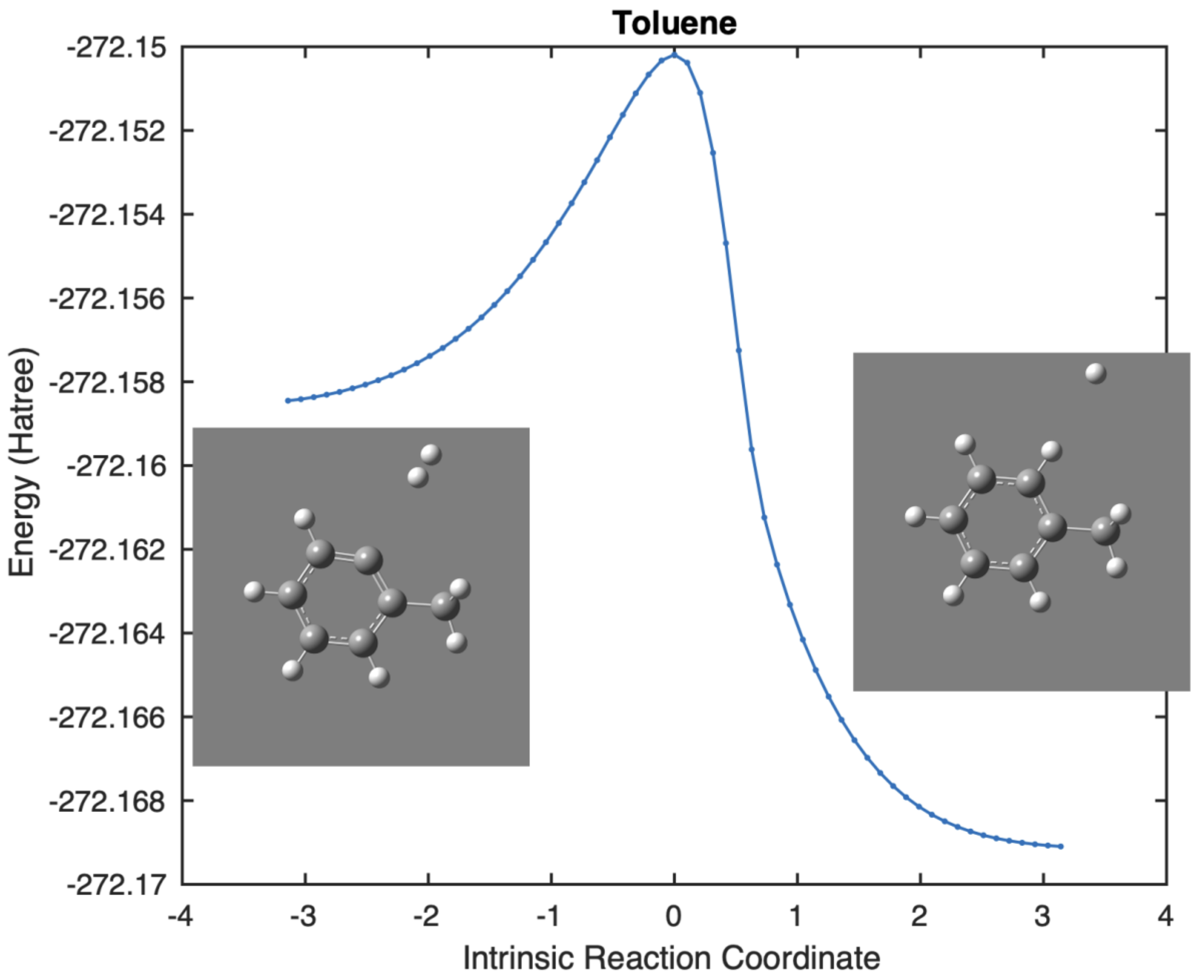

Fig. S19. Intrinsic reaction coordinate scan for toluene radical+H_2 reaction. Molecular images of the terminal points are displayed.

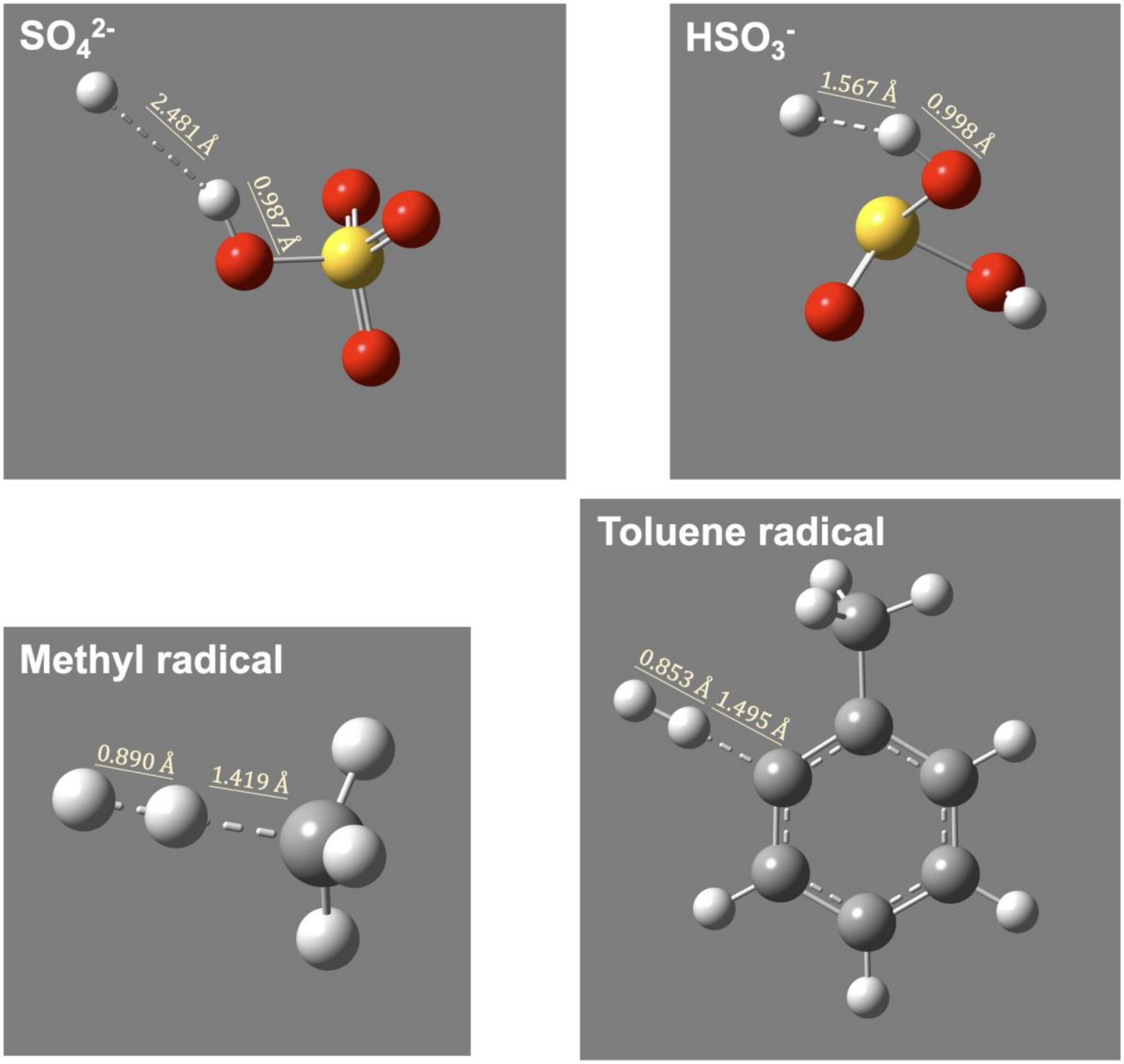

Fig. S20. Transition state structures for the oxidation reactions.

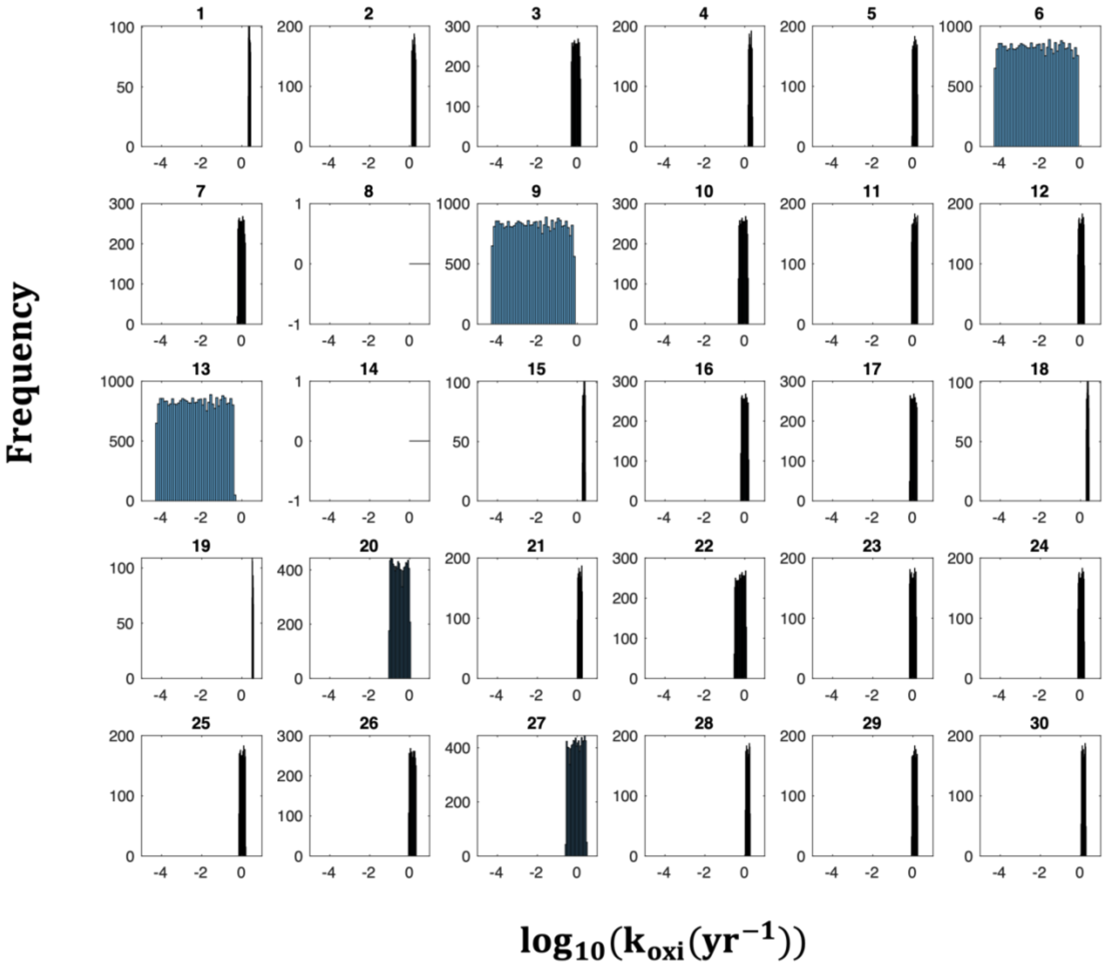

Fig. S21. Distribution of oxidation rates for each sample constrained from Monte-Carlo simulation of the isotope evolution model.

Table S1. Sulfate concentrations in production waters for several sampled wells.

Well	Formation	Depth (m)	SO_4^{2-} concentration (mg/L)
PS–2	Dengying	7730	3250
PT–101	Dengying	5615	969
PS–5	Dengying	5503	1090
PS–13	Dengying	6080	2430

Table S2. Isotopic results in the purification procedure.

Sample	δ^2H_{VSMOW}	1 s.e. δ^2H_{VSMOW}	Δ^2H^2H	1 s.e. Δ^2H^2H
Original gas	-61.99	0.21	121.00	4.67
Purified gas	-69.16	0.24	133.70	5.15

Table S3. Analyses of a sample gas, 'commercial sample 1', as an evaluation of reproducibility.

Analysis date	Sample	δ^2H_{VSMOW}	1 s.e. δ^2H	Δ^2H^2H	1 s.e. Δ^2H^2H
9/17/24	commercial sample 1	-61.99	0.21	121.00	4.67
11/12/24	commercial sample 1	-61.55	0.24	114.97	3.61
5/25/25	commercial sample 1	-63.39	0.31	112.41	4.43

Table S4. Bonding environments for the attacking hydrogen atom. d (X–H) is the distance between attacking hydrogen and the target carbon or oxygen atom (Fig. S20) and υ (X–H stretching) is the frequency for the X–H stretching mode of vibration.

Reactant	d (X–H) Å	υ (X–H stretching) cm^{-1}
SO_4^{2-}	0.987	3435.47
HSO_3^-	0.998	3145.55
Methyl radical	1.419	1129.09
Toluene radical	1.495	1446.16

Table S5. Results of an adsorption experiment.

Sample	δ^2H_{VSMOW}	1 s.e. δ^2H_{VSMOW}	Δ^2H^2H	1 s.e. Δ^2H^2H
Original gas	-61.55	0.24	114.97	3.61
Adsorption free gas	-60.98	0.35	124.73	6.34

Table S6. Qualitative classification of openness of the gas systems based on well reports.

Sample no.	Well ID	Reservoir unit	Relative openness class	Relevant source material
1	HS101	Qixia	Unresolved	Core-plug porosity and permeability records for Hesheng 101
2	RC1	Maokou	Unresolved	No well-specific structural material available in the reviewed collection
3	ZS103	Dengying	Unresolved to weakly open	Zhongshen core-plug porosity and permeability records
4	ZS101	Dengying	Unresolved to weakly open	Zhongshen 101 core-plug porosity and permeability records
5	PT108	Dengying	Partial open-system influence plausible	Regional structural summaries for the Deyang-Anyue and Penglai domains
6	PS5	Dengying	Partial open-system influence plausible	Pengshen 5 core-plug records and regional structural summaries
7	GS136	Dengying	Near-closed to weakly open	Gaoshiti well reports, core observations, and porosity-permeability records
8	GS001-X3	Dengying	Near-closed to weakly open	Gaoshiti well reports, core observations, and porosity-permeability records
9	GS001-H2	Dengying	Near-closed to weakly open	Gaoshiti well reports, core observations, and porosity-permeability records
10	GS102	Dengying	Near-closed to weakly open	Gaoshiti well reports, core observations, and porosity-permeability records
11	GS124	Dengying	Near-closed to weakly open	Gaoshiti well reports, core observations, and porosity-permeability records
12	GS126	Dengying	Near-closed to weakly open	Gaoshiti well reports, core observations, and porosity-permeability records
13	GS133	Dengying	Near-closed to weakly open	Gaoshiti well reports, core observations, and porosity-permeability records
14	GS7	Dengying	Near-closed to weakly open	Gaoshiti well reports, core observations, and porosity-permeability records
15	MX107	Dengying	Weakly to partially open	Moxi logging reports and core descriptions
16	MX13	Dengying	Weakly to partially open	Moxi logging reports and core descriptions
17	MX131	Dengying	Weakly to partially open	Moxi logging reports and core descriptions
18	MX146	Dengying	Weakly to partially open	Moxi logging reports and core descriptions
19	GS101	Dengying	Near-closed to weakly open	Gaoshiti well reports, core observations, and porosity-permeability records
20	MX11	Longwangmiao	Weakly to partially open	Moxi logging reports and core descriptions
21	MX21	Longwangmiao	Weakly to partially open	Moxi logging reports and core descriptions
22	MX23	Longwangmiao	Weakly to partially open	Moxi logging reports and core descriptions
23	MX27	Longwangmiao	Weakly to partially open	Moxi logging reports and core descriptions
24	PS12	Dengying	Partial open-system influence plausible	Pengshen 12 core-plug and lithologic records
25	PT108	Dengying	Partial open-system influence plausible	Regional structural summaries for the Deyang-Anyue and Penglai domains

26	PS5	Dengying	Partial open-system influence plausible	Pengshen 5 core-plug records and regional structural summaries
27	PS13	Longwangmiao	Partial open-system influence plausible	Pengshen 13 core-plug records
28	MX8#	Longwangmiao	Weakly to partially open	Moxi logging reports and core descriptions
29	MX29#	Longwangmiao	Weakly to partially open	Moxi logging reports and core descriptions
30	XT1#	Changxing	Unresolved to weakly open	Xuantan 1 logging summary and regional platform-margin interpretation

Table S7. Comparison of the sink environment for hydrogen systems in the world [24–30].

System	H_2 enrichment	Depth / T	Sink environment	Implication
**Bourakebougou, Mali**	~98% (vol.) H_2; production test ~1500 m^3 H2/day	Main H2-rich reservoir as shallow as ~100 m; free gas mainly in shallow/upper reservoirs, deeper H2 mostly dissolved	Intracratonic dolomitic karst reservoir sealed by dolerite intrusions; published interpretations emphasize sealing and dynamic recharge rather than TSR-type destruction	Strong example of shallow, well-sealed H2 preservation with dynamic recharge
**Bulqizë, Albania**	~84 vol% H2; ≥200 t H2/yr vented from mine	Outgassing at ~500–1000 m below surface; reservoir inferred to be deeply rooted in Jurassic ophiolite	Strongly reducing ophiolitic system with fault-focused gas discharge; sealing/trapping geometry remains uncertain	High H2 can occur in ophiolitic systems where faults focus migration and local reservoirs develop
**Kansas, USA**	Historical Scott #1 and Heins #1 values broadly ~24–80 mol% H2; Sue Duroche #2 initially ~91.7 mol% H2	Wells ~424–770 m; H2–water isotope equilibrium interpreted at ~30–50°C	After basement isolation, H2 decreased strongly; interpreted aquifer/tubing redox reactions and microbial processes consumed or modified H2	Shallow depth alone is insufficient; aquifer sinks and well-completion history strongly affect H2 preservation
**Frog's Leg, Yilgarn Craton, Australia**	19.9–68.7 mol% H2 with abundant methane	Shallow open-hole wells; isotope equilibrium T ~42–57°C implies minimum source depths ~1.4–2.7 km	Mafic–ultramafic / Fe-rich Archean basement; mixed H2–CH4 abiogenic gas system	Supports moderate-temperature mafic/basement systems as H2-rich targets, but depth is inferred source depth
**Samail ophiolite, Oman**	H2-rich hyperalkaline seeps and groundwaters; variable flux	Near-surface to shallow subsurface; low-temperature serpentinization, commonly ≤50°C	Hyperalkaline, reducing peridotite aquifers; microbial H2 consumption can be substantial except along focused fault pathways	Good generation and consumption analogue, not necessarily a trapped accumulation
**Sichuan Basin, this study**	0.10–1.59% H_2	2939–7110 m; 90–175°C	Hydrocarbon-rich, sulfate/H2S-bearing, deep carbonate reservoirs; oxidation and thermal reequilibration likely important	

Data S1. (separate file)

Sample information, chemical composition and isotopic results of gas samples in this study.

Data S2. (separate file)

Compiled published δ^2H values of naturally occurring H_2.

Data S3. (separate file)

Geometries (cartesian coordinates) and calculated frequencies of the transition states in oxidation reactions of H_2 with various oxidants.

Data S4. (separate file)

Kinetic isotope effects (KIE) for isotopologues of H_2 in its oxidation reaction using various oxidants. KIE is defined as the ratio between reaction rate constant of $^1H^1H$ and the specified isotopologue: $KIE = \frac{k_{^1H^1H}}{k_{isotopologue}}$.

Data S5. (separate file)

Estimated rate constants of oxidation using the isotope evolution model and the Monte-Carlo method.
